# Identification of Outer Membrane and Exoproteins of Carbapenem-Resistant Multilocus Sequence Type 258 *Klebsiella pneumoniae*

**DOI:** 10.1371/journal.pone.0123219

**Published:** 2015-04-20

**Authors:** Amanda J. Brinkworth, Carl H. Hammer, L. Renee Olano, Scott D. Kobayashi, Liang Chen, Barry N. Kreiswirth, Frank R. DeLeo

**Affiliations:** 1 Laboratory of Human Bacterial Pathogens, Rocky Mountain Laboratories, National Institute of Allergy and Infectious Disease, National Institute of Health, Hamilton, MT, United States of America; 2 Research Technologies Branch, National Institute of Allergy and Infectious Disease, National Institute of Health, Bethesda, MD, United States of America; 3 Public Health Research Institute Tuberculosis Center, New Jersey Medical School, Rutgers the State University of New Jersey, Newark, NJ, United States of America; Quuen's University Belfast, UNITED KINGDOM

## Abstract

Carbapenem-resistant *Klebsiella pneumoniae* strains have emerged as a cause of life-threatening infections in susceptible individuals (e.g., transplant recipients and critically ill patients). Strains classified as multilocus sequence type (ST) 258 are among the most prominent causes of carbapenem-resistant *K*. *pneumoniae* infections worldwide, but the basis for the success of this lineage remains incompletely determined. To gain a more comprehensive view of the molecules potentially involved in the success of ST258, we used a proteomics approach to identify surface-associated and culture supernatant proteins produced by ST258. Protein samples were prepared from varied culture conditions *in vitro*, and were analyzed by a combination of two-dimensional electrophoresis and liquid chromatography followed by tandem mass spectrometry (LC-MS/MS). We identified a total of 193 proteins in outer membrane preparations from bacteria cultured in Luria-Bertani broth (LB) or RPMI 1640 tissue culture media (RPMI). Compared with LB, several iron-acquisition proteins, including IutA, HmuR, HmuS, CirA, FepA, FitA, FoxA, FhuD, and YfeX, were more highly expressed in RPMI. Of the 177 proteins identified in spent media, only the fimbrial subunit, MrkA, was predicted to be extracellular, a finding that suggests few proteins (or a limited quantity) are freely secreted by ST258. Notably, we discovered 203 proteins not reported in previous *K*. *pneumoniae *proteome studies. *In silico* modeling of proteins with unknown function revealed several proteins with beta-barrel transmembrane structures typical of porins, as well as possible host-interacting proteins. Taken together, these findings contribute several new targets for the mechanistic study of drug-resistance and pathogenesis by ST258 *K*. *pneumoniae *isolates.

## Introduction

Infections caused by Gram-negative bacteria that contain extended spectrum beta-lactamases (ESBLs) have been widespread for decades [1–3]. The rapid acquisition of antibiotic resistance is largely due to the horizontal transfer of antibiotic resistance plasmids among Enterobacteriaceae [4, 5]. More recently, there has been an emergence of carbapenem-resistant (CR) *K*. *pneumoniae* strains as opportunistic hospital pathogens [6, 7]. These normally commensal microbes are resistant to all β-lactam antibiotics and often other important therapeutic agents [8–10]. A CR-*K*. *pneumoniae* strain classified as multilocus sequence type (ST) ST258 is among the most prevalent multidrug-resistant Enterobacteriaceae in hospitals worldwide [11–14]. For ST258, resistance to carbapenem antibiotics is conferred by bla_*kpc*_, the gene encoding plasmid-borne *Klebsiella pneumoniae* carbapenemase (KPC) [6, 15]. ST258 is known to cause respiratory, bloodstream, and urinary tract infections in the US, Brazil, Columbia, Italy, Poland, and Israel [16]. KPC-containing *K*. *pneumoniae* are present in 17.8% of long-term care facilities and 5% of short-term facilities [6]. Long-term stay patients are at the highest risk for *K*. *pneumoniae* infections, especially those with compromised immune systems or that have undergone organ transplants, surgery, or have had device implants [17, 18]. In this patient background, and in the absence of an effective therapeutic agent or treatment, these organisms can cause death [19, 20]. Drug-resistance in *K*. *pneumoniae* is correlated with increased mortality [21]. For example, carbapenem-resistant ST258 strains have been associated with mortality rates ranging from 34–42% [16, 17, 19, 20]. Although progress has been made, including a comprehensive genomic analysis of the ST258 lineage [8], the basis for the success of this strain above and beyond antibiotic resistance remains incompletely determined.

Inasmuch as carbapenems are considered to be the last line of defense against ESBL-containing Gram-negative bacteria, it is imperative to consider alternative treatments and practices to prevent the spread of these microbes. The high mortality rate, increasing burden of resistant organisms, and capacity to acquire resistance to antibiotics rapidly, compels the development of therapies beyond antibiotic use. A passive or active vaccine against CR-*K*. *pneumoniae* is one such potential alternative therapeutic or preventative approach.

A comprehensive view of the surface proteins of important clinical strains (such as ST258) is important for understanding pathogen success and is a step towards vaccine development. Surface proteome studies have been performed with *K*. *pneumoniae* [22–26]. However, the chromosome and plasmids of *K*. *pneumoniae* are somewhat genetically diverse and there is little or no information about the surface proteins of ST258. To address this deficiency in knowledge, we used a proteomics approach to identify surface-exposed and secreted proteins produced by ST258 clinical isolates.

## Materials and Methods

### Bacterial cultures

Clinical isolates were selected from a repository maintained at the Public Health Research Institute TB Center, Rutgers New Jersey Medical School. *K*. *pneumoniae* isolates used in the current studies were classified as ST258 (i.e., isolates 30660 and 30684). The complete genome sequences for these isolates are available in the GenBank database (isolate 30660 = NJST258_1, GenBank NCBI accession number CP006923; isolate 30684 = NJST258_2, GenBank NCBI accession number CP006918). All isolates were grown at 37°C with aeration. Cultures were inoculated at 5 × 10^3^ cells/mL, except for those grown in M63 minimal media, in which cultures were inoculated with 5 × 10^5^ cells/mL. Luria-Bertani broth (LB), RPMI 1640 medium buffered with HEPES and supplemented with L-glutamine (RPMI), super optimal broth with catabolite repression (SOC), trypticase soy broth (TSB), brain-heart infusion (BHI) medium, Dulbecco’s Modified Eagle Medium (DMEM) with 10% heat-inactivated fetal-bovine serum (FBS), and M63 minimal medium, were used to culture bacteria.

### Outer membrane extractions

Isolate 30660 was cultured to mid-exponential or stationary phase of growth in 200 mL LB or RPMI. Bacteria were collected at 10,000 × *g* for 30 min, resuspended in lysis buffer (50 mM Tris pH 8.0, 10 μg/mL DNase I, 2 μg/mL RNase A, protease inhibitor cocktail) and lysed by three passes through a French press. Bacterial debris was removed at 4000 × *g* and outer membranes were enriched from the supernatant as described previously [27], with modifications. Briefly, membranes were pelleted by ultracentrifugation (100,000 × *g*) and washed sequentially with 25 mM Tris, pH 8.0, containing 0.3 M sucrose and 2.5 mM EDTA (TSE buffer), 2% N-lauroylsarcosine sodium salt (Sigma-Aldrich) in 50 mM Tris, and 0.1 M sodium carbonate in 50 mM Tris. Purified outer membranes were resuspended in 1 mL 0.1× TSE buffer and were stored at -20°C.

### Enzymatic assays

Subcellular fractions were analyzed for the presence of markers beta-galactosidase (cytoplasm), succinate dehydrogenase (inner membrane), and 3-deoxy-D-*manno*-oct-2-ulosonic acid or KDO (outer membrane) (S1 Fig). Fractions were adjusted to 10 μg protein content. Beta-galactosidase activity was measured using the Beta-galactosidase Enzyme Assay System (Promega) according to the manufacturer’s instructions. Succinate dehydrogenase activity was measured using the Succinate Dehydrogenase Activity Colorimetric Assay Kit (Sigma-Aldrich) per the manufacturer’s instructions, except samples were incubated on ice in SDH Assay buffer for 6 h prior to starting the assay. The concentration of KDO was determined as described previously [28].

### Enrichment of proteins in culture supernantants (exoproteins)

ST258 isolate 30684 was cultured to early stationary phase of growth in 100 mL TSB or M63 minimal media supplemented with 200 μM FeSO_4_ and 0.4% glycerol. The extent of cytolysis during standard culture (above) was tested using the Live/Dead BacLight Bacterial Viability Kit (Molecular Probes) as per the manufacturer’s instructions. Fluorescence microscopy was performed with an LSM 5 live microscope and images were captured with an Axio HRC camera and Axio Vision SE64 Rel 4.9 software (S2 Fig). Bacteria were centrifuged at 4000 × *g* and supernatants were aspirated and clarified by using 0.2 μm filters. Exoproteins were concentrated by centrifugal filtration with 3 kDa-cutoff Amicon ultra filtration tubes (EMD Millipore) to a final volume of 1 mL and stored at -20°C. Protein concentrations were determined by using the bicinchoninic acid assay (BCA) (Pierce) as previously described [29].

### Protein preparation and separation

For one-dimensional SDS-PAGE, samples were boiled at 95°C for 5 min in Laemmli sample buffer (Bio-Rad) and resolved using pre-cast Criterion 10.5–14% polyacrylamide gels in a Tris-glycine-SDS buffer system. Proteins were detected by G250 Coomassie Blue stain (Sigma-Aldrich) or SYPRO Ruby protein stain (Life Technologies).

Proteins were extracted from outer membranes by methanol-water-chloroform extraction as previously described [30]. Proteins were re-solubilized in rehydration buffer (10 mM Tris, 6 M urea, 2 M thiourea, 1% ASB-14, 2% CHAPS, 50 mM DTT) and 200 μg of protein was passively rehydrated into 11-cm, pH 3–10 IPG strips (Bio-Rad) for 15 h at ambient temperature. Isoelectric focusing was performed as a step gradient that terminated at 8000 V for 30,000 volt-h with a Protean IEF cell (Bio-Rad). IPG strips were placed onto Criterion 10.5–14% Tris-HCl IPG+1 pre-cast gels and proteins were separated in the second-dimension by SDS-PAGE at a constant 50 mA for 2 h. Protein spots were detected under UV-light at 206 nm following staining with SYPRO Ruby.

### Preparation of protein for mass spectrometry

Gel plugs (1.2 mm) from 2D-gels were washed twice with ultrapure water, twice with 50 mM ammonium bicarbonate/ 50% acetonitrile (AcCN), and once with 100% AcCN. Reduction-alkylation reactions were performed in-gel with 60 mM DTT at 65°C for 30 min, and with 60 mM iodoacetamide at ambient temperature in the dark for 1 h. The above wash steps were repeated and proteins were digested in-gel with 10 ng/μL porcine trypsin (Sigma-Aldrich) overnight at 30°C. Tryptic peptides were extracted from the gel plugs with formic acid for 30 min at 37°C, followed by lyophilization in SUN-SRI microsampling vials with a SpeedVac. Nano-liquid chromatography coupled to tandem mass spectrometry (LC-MS/MS) was performed as previously described [31]. Briefly, lyophilized samples were resuspended in solvent A (0.1% formic acid, 2% AcCN), and peptides were separated on a ProXeon Easy-nLC I multidimensional liquid chromatograph with a linear gradient of solvent A and solvent B (0.1% formic acid, 97.9% AcCN). Bound peptides were nanosprayed in-line with an LTQ-Orbitrap mass spectrometer, and data were acquired and peptides were sequenced by Xcalibur 2.1 software. MASCOT 2.4 software (Matrix Science) was used to query peptide sequences against a concatenated target/decoy database containing common contaminants (cRAP.fasta, The Global Proteome Machine) and protein sequences from Uniprot KB/TrEMBL for *E*. *coli* (strain K12), *Salmonella* (strain LT2), and ST258 *K*. *pneumonia* isolate 30660 (NJST258_1, GenBank NCBI accession number CP006923). All sequences were downloaded 6/2014. Data were parsed using ProteoIQ v 2.7 (PREMIER Biosoft) with an initial peptide FDR of 0.5%. Peptides were filtered further using the Protein Prophet algorithm as implemented in ProteoIQ to include only those with peptide and protein group probability cutoff scores of 0.95 and 0.99, respectively. Analyses of 2 spectra per peptide / 2 peptide per protein minimums resulted in a calculated protein FDR of 0.

### *In silico* modeling of uncharacterized CR-*K*. *pneumoniae* proteins

The amino acid sequences of uncharacterized proteins that localized to the outer or periplasmic membrane, or those of unpredicted localization were submitted to the web-based structural modeling server Phyre [32, 33]. A representative model was chosen based on confidence score, homology, insertions/deletions in sequence, and the alignment accuracy assessment as outlined previously [32]. The structural data files (.pdb) for the modeled proteins were uploaded onto the web-based membrane prediction server TM-DET [34]. First-Glance in Jmol viewer (Eric Martz, http://bioinformatics.org/firstglance/fgij/) was used to capture high resolution images of the predicted structures.

## Results

### Identification of outer membrane proteins

**Fig 1:**
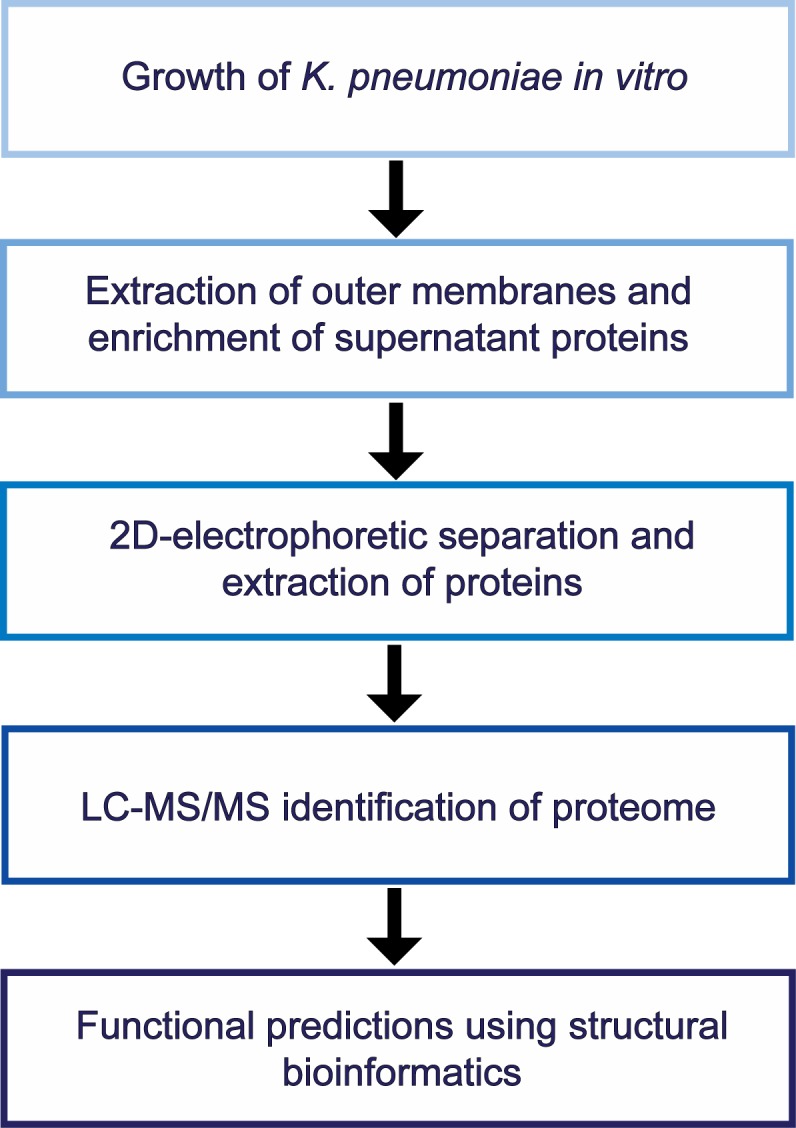
Workflow for the identification of *K*. *pneumoniae* surface and secreted proteins.

**Fig 2:**
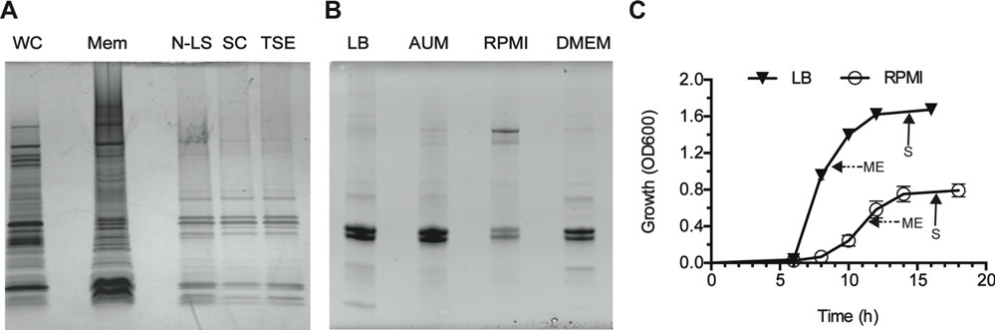
Enrichment of *K*. *pneumoniae* outer membrane proteins. (A) Protein profile at each step in the outer membrane (OM) extraction process. WC = whole cell lysate from *K*. *pneumoniae* isolate 30660, Mem = total membrane fraction, N-LS = N-lauroylsarcosine treatment, SC = sodium carbonate wash, TSE = Tris-sucrose-EDTA. (B) Comparison of outer membrane proteins in different media. AUM = artificial urine media. (C) Growth of *K*. *pneumoniae* isolate 30660 in LB or RPMI. Arrows with solid lines indicate sample collection at mid-exponential (ME) phase of growth and arrows with dashed lines indicate sample collection at stationary (S) phase of growth.

We used a standard proteomics approach to identify proteins present on the surface of a representative ST258 clinical isolate (30660) (Fig 1). We selected this particular clinical isolate because the genome is closed and fully annotated, and it is representative of widespread ST258 CR*-K*. *pneumoniae* strains [8]. The expression of outer membrane proteins was evaluated using multiple culture conditions to expand the protein expression profile. Expression patterns were similar for bacteria cultured in LB, AUM, and DMEM (Fig 2B). By comparison, outer membrane protein expression was distinct for those cultured in RPMI, perhaps due to the lack of iron in RPMI (Fig 2B). In the end, we chose LB and RPMI as culture media for subsequent proteomics studies.

**Fig 3:**
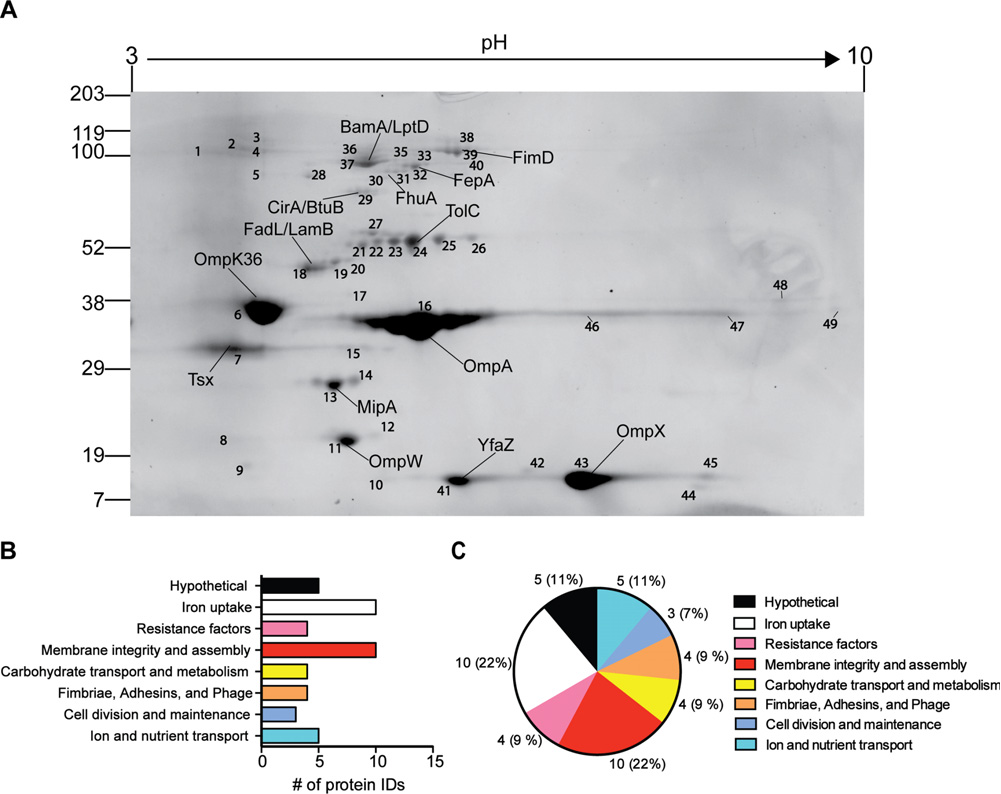
Identification of *K*. *pneumoniae* outer membrane proteins by 2DE. (A) Two-dimensional map of outer membrane proteins from isolate 30660 cultured to stationary phase of growth in LB. Numbers refer to the extracted spots that were processed for identification by LC-MS/MS. The pI and molecular weight markers are shown on the top and right, respectively. (B) Categorical representation by function of predicted outer membrane proteins. (C) Categorical representation by function of predicted outer membrane proteins.

Outer membrane proteins were resolved by 2-dimensional electrophoresis (2DE) and proteins spots were extracted from gels and analyzed by LC-MS/MS (Fig 3A). Positive protein identifications were defined as those having a mascot score greater than 100, at least 10% sequence coverage, and a minimum of 2 peptide hits (S1 Table). We identified 193 proteins under all conditions tested, including 45 predicted to be localized to the outer membrane (23%), 100 in the cytoplasm (52%), 21 in the cytoplasmic membrane (11%), 4 exported (2%), 7 in the periplasm (4%), and 16 of unknown localization (8%). We used the functional annotation of protein homologs in the Uniprot database to assign a putative function to each protein. Of the 45 proteins predicted to be in the outer membrane, 10 are likely to be involved in membrane assembly and integrity, 10 in iron uptake, 5 have no known function, 5 in ion or nutrient transport, 4 each in antimicrobial resistance, carbohydrate metabolism and transport, or fimbrial assembly and adhesions, and 3 in cell division and maintenance. (Fig 3B and 3C). Of the 16 proteins of unknown localization, 8 of these were proteins with no known function.

### Impact of culture medium on membrane protein expression

**Fig 4:**
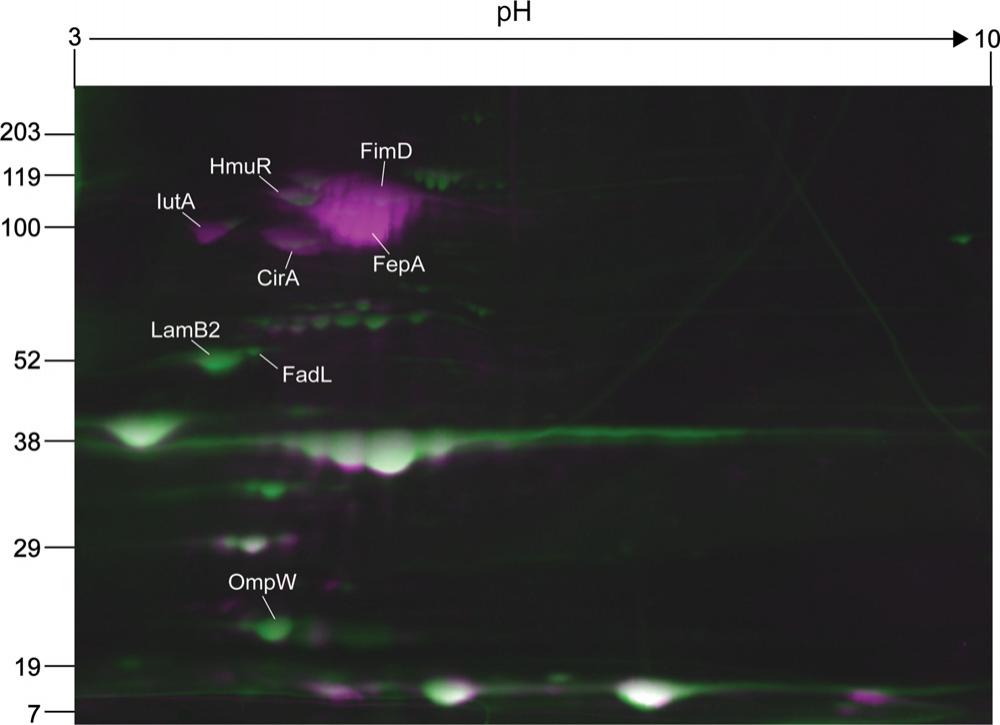
Comparison of outer membrane protein expression in LB and RPMI growth media. Representative 2D gels of stationary-phase outer membrane proteins expressed in LB (green) or RPMI (purple) were overlayed in Samespots software (Nonlinear Dynamics). White spots indicate colocalized proteins.

To compare surface protein expression between growth phases or culture media, protein spots from each condition were quantified using Samespots software (Nonlinear Dynamics). Spot intensity was normalized to the background of each individual gel and averaged among three gels per growth condition. For this analysis, we focused on proteins with a >2 fold-difference in spot abundance and a *P*-value < 0.05. Comparison of outer membrane proteins expressed in LB during mid-exponential and stationary phases of growth revealed no significant differences (data not shown). On the other hand, there were significant differences in ST258 protein expression levels between LB and RPMI during stationary phase of growth (Fig 4). The fatty acid transport protein, FadL, and the porins, LamB1 and OmpW, were significantly more abundant on the bacterial surface in LB culture compared with that in RPMI, whereas the fimbrial subunit, FimD, and the iron receptors, IutA, HmuR, CirA, and FepA, were more highly expressed during culture in RPMI (Table 1). Iron receptors have been shown to play an important role in survival of *K*. *pneumoniae* during mammalian infection [35, 36], and iron levels directly influence gene expression through the transcriptional activator Fur [37, 38]. The surface expression of several iron receptors during growth in RPMI (a low-iron culture medium) could be similar to that of the iron-restrictive environment of the mammalian host.

**Table 1:** Comparison of outer membrane protein abundance in LB and RPMI.

Protein	ANOVA (*p*-value)^a^	Fold-difference (LB/RPMI)^a^	Theoretical pI^b^	Theoretical MW^b^	LB^c^	RPMI^c^
IutA	0.002	-3.8	4.23	93	9.60E+05	3.64E+06
HmuR	0.035	-2.4	4.94	99	4.35E+06	1.04E+07
CirA	0.002	-6.3	4.84	87	1.33E+06	8.38E+05
FepA	0.003	-15.3	5.31	94	1.97E+06	3.01E+07
FimD	0.035	-3.7	5.31	101	4.94E+06	1.85E+07
LamB2	0.001	5.4	3.96	50	6.53E+06	1.22E+06
FadL	0.008	4.0	4.62	55	5.84E+05	1.46E+05
OmpW	0.006	4.9	4.51	21	9.06E+06	1.84E+06

Analysis was performed using protein spots from three individual 2D-gels per growth condition.

^a^) Fold differences and *p*-values were determined by Samespots software (Nonlinear Dynamics).

^b^) Theoretical pI and molecular weight were determined by linear regression in Samespots.

^c^) Average normalized volumes of protein spots. Protein volumes were normalized to the background of each 2D gel by Samespots.

### 1D-separation and MS-identification of proteins in culture supernatant

**Fig 5:**
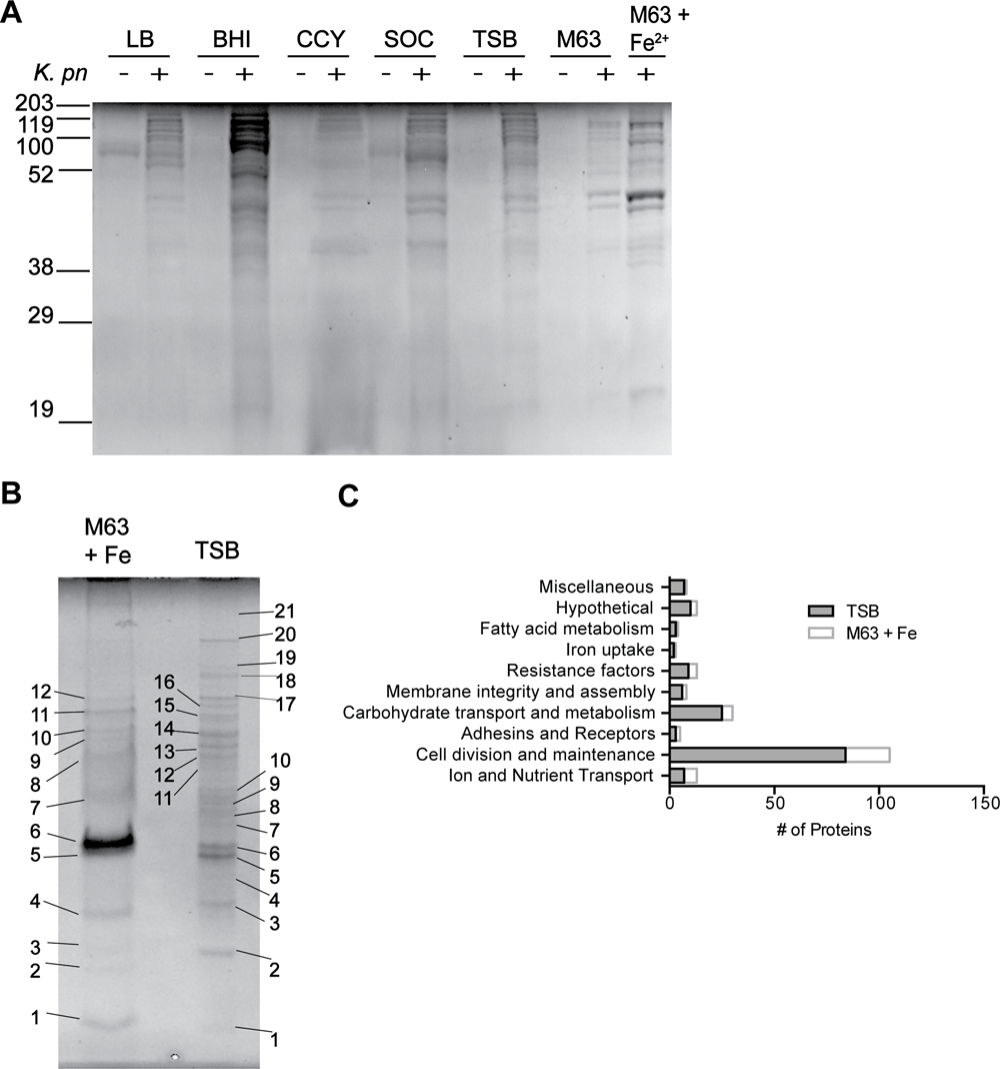
Enrichment of exoproteins. (A) Culture supernatant proteins from *K*. *pneumoniae* at late-exponential phase of growth in M63 minimal media supplemented with 0.4% glycerol and 200 μM FeSO4 or in TSB. Numbers refer to the extracted spots that were processed for identification by LC-MS/MS. (B) Exoproteins from culture supernatants at late-exponential phase of growth for *K*. *pneumoniae* isolate 30684 in different culture media. (+) = bacteria present in culture; (−) = media without bacteria. (C) Categorical representation by function of proteins identified from spent media.

During *K*. *pneumoniae* infections, the host interacts with bacterial molecules that are actively and freely secreted or those shed from the bacteria. Previous studies reported the presence of *K*. *pneumoniae* exoproteins in supernatants from cultures grown in minimal media supplemented with glycerol or in LB [39, 40]. However, such an analysis has not been conducted for ST258. Thus, we investigated culture conditions for accumulation of proteins in spent culture media (i.e., exoproteins, which includes proteins that are freely secreted, those shed from the surface, and those nonspecifically released from dead cells) by isolate 30684, another ST258 isolate for which there is a closed and anotated genome [8]. The isolate 30660 has a mucoid phenotype, which includes being somewhat resistant to pelleting by centrifugation. For this reason, we selected a non-mucoid ST258 isolate (30684) that is more amenable for culture supernatant collection for these assays. Maximal protein accumulation in media occurred in late exponential phase with all growth media tested (data not shown), albeit the level of protein was low. To identify culture media optimal for protein secretion/accumulation, we used SDS-PAGE to analyze proteins present in BHI, CCY, LB, M63, SOC, and TSB supernatants after bacterial culture (Fig 5A). Proteins produced/shed in LB, SOC, and TSB were qualitatively similar. Concentrated BHI supernatants were viscous with media contaminants and were therefore unacceptable for subsequent mass spectrometry analysis. Compared to the other culture media (except BHI), more exoproteins were present in M63 supplemented with FeSO_4_ (Fig 5A). The electrophoretic pattern of proteins from TSB culture supernatants was consistently clear of media contaminants compared to LB, SOC, and CCY. Based on these data, we chose to analyze exoproteins from TSB and M63 cultures grown to late-exponential phase.

Exoproteins from bacteria grown in TSB or M63 were resolved by SDS-PAGE, extracted, trypsin-digested, and analyzed by LC-MS/MS (Fig 5B and S2 Table). Of the 177 proteins identified from spent media, 114 are predicted to localize to the cytoplasm, 17 to the outer membrane, 30 to the periplasm, 3 to the cytoplasmic membrane, 1 to extracellular space, and 12 are of unknown localization. Functional characterization of these proteins indicates that 95 are likely to be involved in cell division or maintenance, 27 in carbohydrate transport and metabolism, 11 have no known function, 10 are associated with antimicrobial or superoxide radical resistance, 8 in protein export or membrane assembly and integrity, 10 in ion or nutrient transport, 3 in iron uptake, 3 are likely to be adhesins or receptors, and 3 are likely to be involved in fatty acid metabolism (Fig 5C). Several proteins that likely protect *K*. *pneumoniae* from environmental stress were identified in spent media, including superoxide-resistance factors (SodB, TrxB, TrxC) and hydrogen peroxide-resistance proteins (KatE, AdhE, AhpF). Additionally, several proteases were shed into the media, including the membrane metalloproteases PmbA and YhjJ.

### Structure of proteins with uncharacterized function

**Fig 6:**
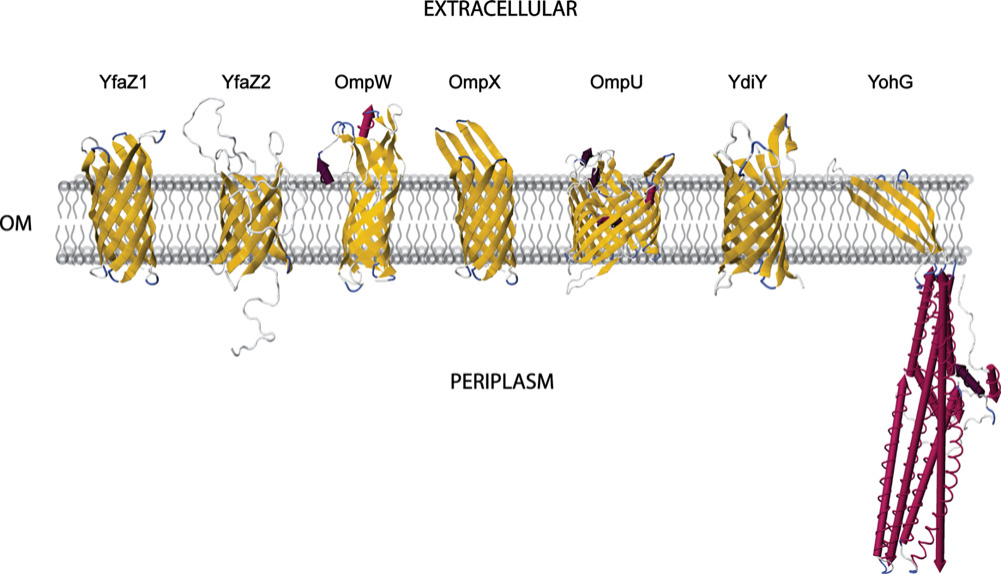
Predicted structures of porin-like hypothetical proteins. The amino acid sequence for each hypothetical membrane protein was modeled *in silico* using Phyre. A representative three-dimensional structure was selected for each porin-like protein and the orientation of each protein with respect to the membrane was predicted using TM-DET. First Glance Jmol Viewer was used to capture high resolution images of each structure, and these images were incorporated manually into a representative outer membrane. The outer membrane (OM) is in grey, beta sheets are yellow arrows, alpha helices are purple or magenta rockets, white lines are coiled regions, and blue lines are turns.

Several proteins identified from outer membrane preparations or culture supernatants had no predicted function. Inasmuch as structural elements of proteins can allude to function, we used amino acid sequences of these uncharacterized proteins in combination with Phyre (a web-based protein structure prediction method) to model three-dimensional (3D) structure. Phyre generates consensus secondary sequences and structures that are then used to generate 3D models of the query protein. These protein structures are based on template proteins that have been submitted to the RCSB protein database as crystal structure or NMR data. Despite low amino acid sequence identity of several of the *K*. *pneumoniae* proteins with proteins used as structural templates, we determined putative structures for 18 outer membrane proteins with greater than 96% confidence (Table 2). The predicted protein structures were analyzed further for their orientation within the outer membrane using the prediction server TM-DET (Fig 6). Notably, six of these proteins, YfaZ1, YfaZ2, OmpW, OmpX, OmpU, and YdiY, had structures highly indicative of outer membrane porins, and YohG is likely an outer membrane efflux protein (Fig 6). Consistent with these findings, *K*. *pneumoniae* OmpX (also known as OmpK17) was shown previously to confer partial resistance to bacteriocin 28b when expressed on the surface of *E*. *coli* [41]. Additionally, our structural analysis suggests that several of the unknown *K*. *pneumoniae* proteins play a role in the interaction with eukaryotic cells (Lectin, DaaE adhesin, Plexin B2, Lipocalin) or interfere with immune-signaling (IgG-binding protein, STAT-4). Thus, our *in silico* anaylsis provides insight into the function of several previously uncharacterized surface molecules that may contribute to the success of the ST258 lineage.

**Table 2:** Phyre structural predictions for *K*. *pneumoniae* hypothetical proteins.

Uniprot ID	Protein	Length (aa)	1^st^ Hit PDB	Protein Template	Conf	Id	2^nd^ Hit PDB	Template	Conf	Id
W8VNC5	OmpU	381	1osm	OmpK36	100	16	3nsg	OmpF	100	15
G8VZE7	Hyp	313	3dkt	Maritamacin	99.5	14	2e0z	Virus-like particle	97.7	18
G8VZH0	Hyp	1007	2bcm	DaaE adhesin	31	25	2khr	MbtH	24.3	50
W8VEP4	OmpX	172	1qj8	OmpX	100	82	3qra	Ail	100	45
W8VLL9	OmpW	224	2f1t	OmpW	100	84	2x27	OprG	100	49
W8VBM4	YbjP	171	4hzb	Tae3 T6SS immunity	96.7	13	2cw9	Tim44 domain	28.9	16
W8VBT5	YedD	144	4hwm	YedD	100	97	1ggl	CRBP, lipocalin	23.2	13
W8VPI0	YfaZ	181	1qj8	OmpX	99.8	16	3qra	Ail	99.7	12
W8VA83	YgaU	149	2l9y	Lectin	99.7	36	2djp	SB145-LysM domain	99.5	24
W8UYP1	YohG	479	1wp1	OprM	100	26	3pik	CusC	100	25
W8URS1	YiaF	250	1fc2	Igg-binding protein	66.2	36	1lp1	Igg-binding, Protein Z	62.9	38
W8VJJ2	VacJ	285	4e71	Rho-binding protein, Plexin B2	37.9	47	3kuz	Ubiquitin-like Plexin C1	36	33
W8VCT6	YajG	181	2iqi	Anti-codon binding	97.6	12	2k7r	Primosomal protein DnaI	85.6	20
W8VJF0	YfhG	235	1bgf	STAT-4 txn factor	85.7	16	4c47	SadB trimeric lipoprotein	57.9	27
W8V2D5	RlpA	382	4avr	Unknown PA4485	100	40	1x60	Peptidoglcan-binding protein	99.6	24
W8V0V3	YncD	701	3qIb	Enantio-pyochelin receptor	100	19	2grx	FhuA	100	24
A6T9R7	Hyp.	68	2k57	Sm-like ribonucleoprotein	99.9	25	2rb6	Sm-like ribonucleoprotein	99.9	45
W8UZI7	Slp	215	4f54	Vpi-5482	67	13	2vbc	NS3 hydrolase/protease	46.1	18
W8V038	YdiY	252	2k0I	OmpA	97.4	14	2iwv	OmpG	97.1	15
W8V807	YfaZ2	180	3qra	Ail	99.6	21	2k0I	OmpA	99.5	18
A6T8N5	YdgH	316	2jna	YdgH	100	27	4evu	YdgH	100	85
A6T9R8	YncE	353	3vh0	YncE, DNA binding protein	100	80	3u4y	Unknown dtox_1751	100	13
A6TCL1	YfiB	127	2i26	Rv0899 membrane protein	100	36	3td4	Omp38	100	37
W8VKY8	YnfB	114	2y6x	Photosystem II protein	33.5	30	2kmf	Photosystem II protein	33.1	25

Phyre was used to model proteins of unknown function. Two structural models with the highest alignment accuracies and their respective protein templates are listed for each query. The RCSB Protein database indentification (PDB) is given for each template. Hyp, hypothetical; Conf, confidence; Id, identity; aa, amino acids.

## Discussion

Here we identified outer membrane proteins and exoproteins of CR-*K*. *pneumoniae* clinical isolates representative of the widespread ST258 lineage. Although a number of these *Klebsiella* membrane proteins have been identified previously, such as OmpA, OmpK36, OmpW, OmpX, Tsx, LamB1, and TolC [23, 25, 42], we report those made by ST258, which includes many uncharacterized surface proteins. The 2DE-staining pattern of our protein samples is remarkably similar to that of Molloy et al., who used a MALDI-TOF peptide mass fingerprinting approach to identify proteins. However, we identified a greater number of proteins on the bacterial surface by comparison. The increased protein detection is presumably due to greater sensistivity of LC-MS/MS compared with MALDI-TOF peptide mass fingerprinting, although we cannot rule out other variables such as differences in sample preparation or culture medias. Not surprisingly, many exoproteins identified by our study overlap with proteins found in *K*. *pneumoniae* outer membrane vesicles [24]. However, 21 of the outer membrane and lipoproteins—namely, BamA, CirA, CutF, FimA, FimF, FimI, FitA, Fiu, HmuR, LptE, OsmE, OprD, ScrY, Slp, YajG, YdiY, YfaZ2, YedD, YiaF, YnfB, and YtfM—have not been identified previously in *K*. *pneumoniae* proteomic studies. Interestingly, the resistance porin OmpK35 was not identified in our analysis, which is consistent with the presence of a stop codon in the *ompK35* gene in this ST258 isolate [8].

Several proteins with hypothetical or unknown function were identified by our study, and we gained insight into the putative functions of these proteins using the web-based structural modeling server, Phyre. The *in silico* analysis revealed that YfaZ1, YfaZ2, OmpW, OmpX, YohG, and YdiY are highly homologous to porins based on secondary structure, and recent work has shown that changes in the expression of the *K*. *pneumoniae* porins OmpK35 and OmpK36 can alter sensitivity to antibiotics [43, 44]. Inasmuch as the porins identified in this study are highly conserved in Enterobacteriaciae, it is possible they contribute to the observed antibiotic resistance phenotype.

In addition to antibiotic resistance mechanisms, the survival of *K*. *pneumoniae* isolates is associated with the expression of multiple iron acquisition systems [35, 36]. Here we compared outer membrane protein expression in rich LB media with expression in the iron-restrictive growth media RPMI. Low-iron conditions not only induced higher expression of several known iron transporters (IutA, FepA, and CirA), but also revealed several iron acquisition molecules (FoxA, FhuD, FitA, YfeX, HmuR, and HmuS) that were uniquely expressed in RPMI compared to growth in LB. Further studies are needed to better understand the role—if any—played by these molecules in the success of ST258 as a human pathogen.

Our method of outer membrane extraction did not exclusively enrich for outer membrane proteins. Indeed, while markers of the cytoplasm and inner membrane were less abundant in outer membrane preparations, they were not removed completely (S1 Fig). Thus, there is some cross-contamination of fractions and mislocalization of proteins. This is likely due to tight associations between outer membrane proteins and proteins from other subcellular compartments of the cell, or from contamination by cytoplasmic membrane proteins that span multiple membranes and do not completely dissociate by treatment with *N*-lauroylsarcosine and sodium carbonate. While the PsortB 3.0 algorithm has been shown to be accurate for predicting subcellular localization [45], some proteins cannot be accurately predicted with this algorithm alone. However, annotations based on homologous proteins indicate that many of these proteins localize to outer membranes or to the periplasmic space.

LC-MS/MS is a sensitive approach and detects the vast majority of proteins present in a sample. However, as with any technique that relies upon trypsin for peptide generation, it is possible some proteins may not be detected at significant levels due to a lack of sites for trypsin digestion. Another limitation of 2DE for the identification of a protein mixture is that basic proteins do not always resolve well during isoelectric focusing, and thus do not always get extracted from the second-dimension gel for MS identification. That said, we identified several outer membrane proteins in the pI 8–11 range. Furthermore, all of the high pI outer membrane proteins that had been previously identified in a mixed protein analysis (2DE-independent) of *K*. *pneumoniae* outer membrane vesicles were also identified by our approach [24].

There are few published reports of proteins secreted by *K*. *pneumoniae*. Early studies using minimal media suggested there is pullulanase and microcin activity in culture supernatants [39, 40]. More recent studies described proteolytic activity in culture supernatants, indicative of elastase [46] and an immunogenic factor of 30–50 kDa [47]. A major limitation of these previous studies was lack of protein sequence identification to confirm the observations. Our study fills this gap in knowledge. Specifically, we used a panel of growth media to test for conditions that enrich for culture supernatant proteins. In addition, we employed a highly sensitive LC-MS/MS approach to identify exoproteins. One caveat of our approach is that the method does not distinguish between proteins released into the media following cytolysis, those shed from the surface non-specifically, and those actively secreted from live bacteria. We determined there was limited bacterial lysis during our culture conditions, indicating that the majority of cytosolic proteins in culture supernatants have not accumulated due to cytolysis (S2 Fig).

*In silico* analysis of proteins with no known function revealed that several of them are putative porins that may contribute to the multi-drug resistance phenotype of these clinical isolates. Further studies of these proteins is necessary to elucidate their possible role in antibiotic resistance and pathogenesis. Additonally, the unique outer membrane proteins identified in this study provide several new potential targets in our efforts to design better therapeutics and/or vaccines for *K*. *pneumoniae* infections.

## Supporting Information

S1 Fig Subcellular enrichment of specific markers during outer membrane extraction. (A) Beta-galactosidase activity (cytoplasm). (B) Succinate dehydrogenase activity (cytoplasmic membrane). (C) KDO concentration (outer membrane). All samples were adjusted to 10 micrograms of protein.
(TIF)

S2 Fig Viability of isolate 30684 grown in TSB or M63 media. 10^8^ bacteria cultured to late-exponential phase of growth were stained with a 1:1 ratio of propidium iodide (red) and Syto 9 (green) using the Live/Dead BacLight Bacterial Viability Kit (Molecular Probes). (A) Images of bacteria were captured by fluorescence microscopy. (B) Fluorescent bacteria were counted manually to determine the % dead in each bacterial preparation.
(TIF)

S1 Table LC-MS/MS protein identifications from outer membrane preparations. (DOCX)

S2 Table LC-MS/MS protein identifications from spent media. (DOCX)
