# Supplementary figures and images for: Identification of Outer Membrane and Exoproteins of Carbapenem-Resistant Multilocus Sequence Type 258 Klebsiella pneumoniae
Source: PLoS One. 2015 Apr 20;10(4):e0123219. doi: 10.1371/journal.pone.0123219 (PMC4404324; doi:10.1371/journal.pone.0123219)

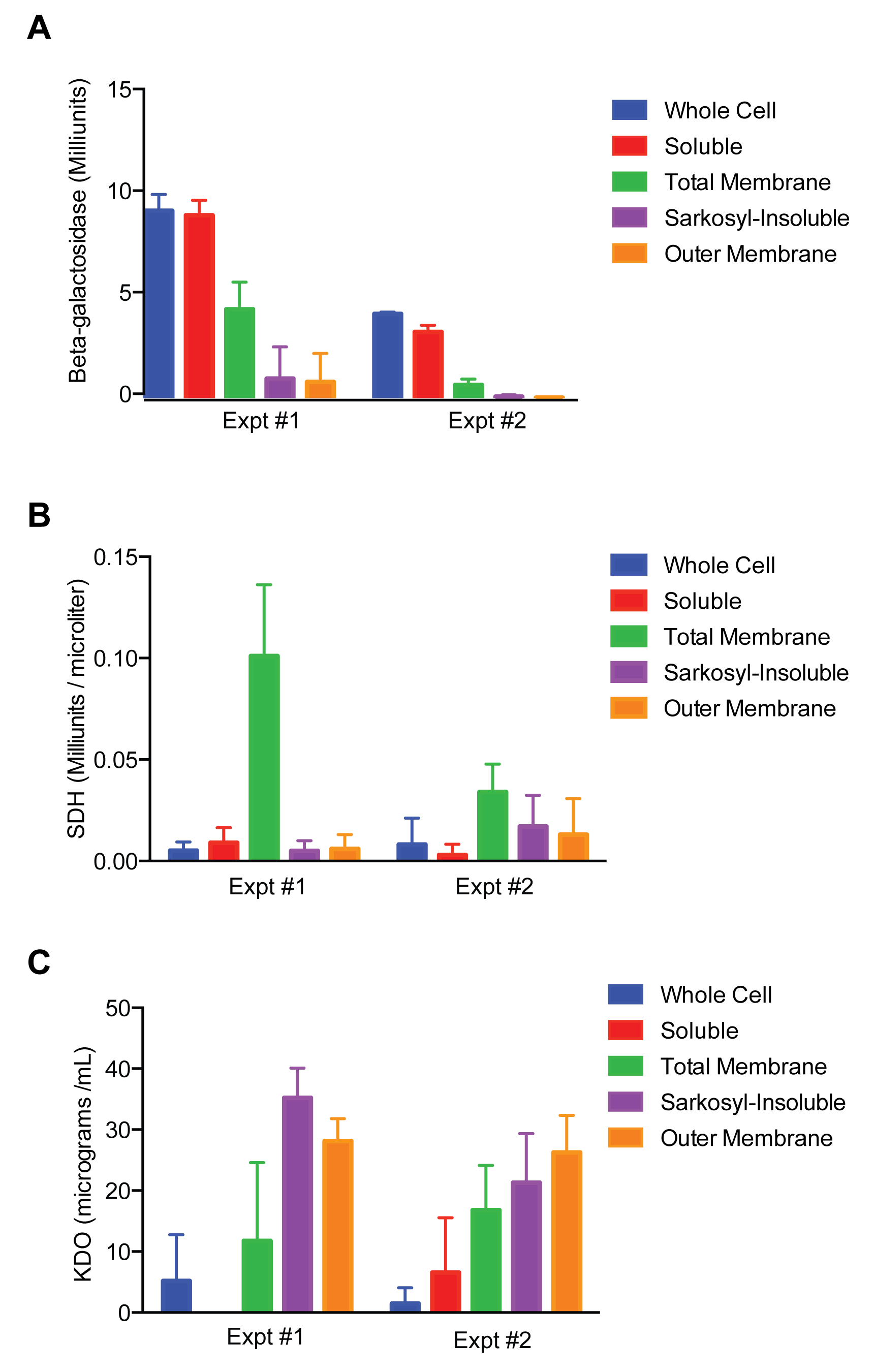

Supplement: S1 Fig — (A) Beta-galactosidase activity (cytoplasm). (B) Succinate dehydrogenase activity (cytoplasmic membrane). (C) KDO concentration (outer membrane). All samples were adjusted to 10 micrograms of protein. (TIF) [file pone.0123219.s001.tif]

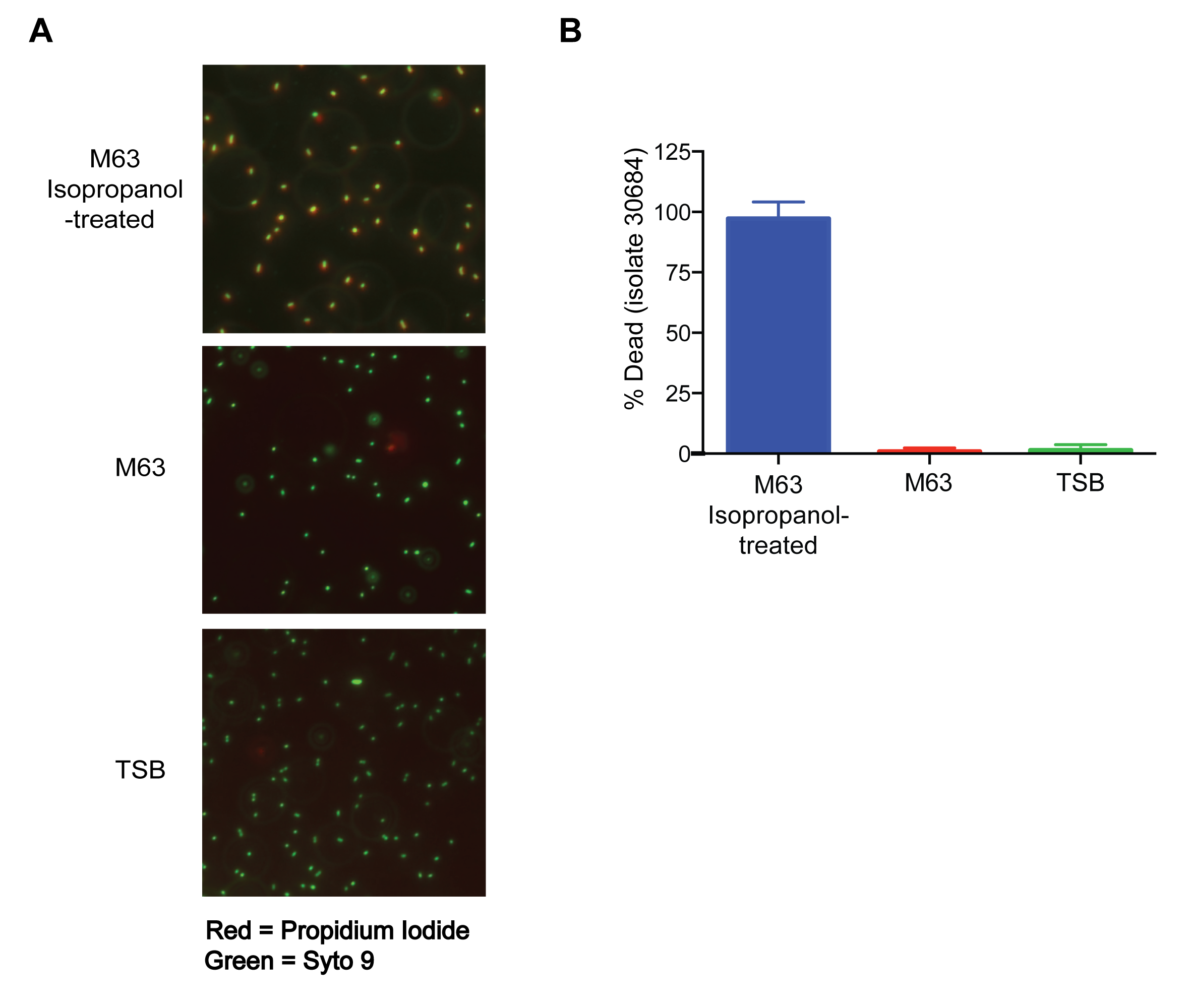

Supplement: S2 Fig — 108 bacteria cultured to late-exponential phase of growth were stained with a 1:1 ratio of propidium iodide (red) and Syto 9 (green) using the Live/Dead BacLight Bacterial Viability Kit (Molecular Probes). (A) Images of bacteria were captured by fluorescence microscopy. (B) Fluorescent bacteria were counted manually to determine the % dead in each bacterial preparation. (TIF) [file pone.0123219.s002.tif]
