# Supplementary material for: Identification of Outer Membrane and Exoproteins of Carbapenem-Resistant Multilocus Sequence Type 258 Klebsiella pneumoniae
Source: PLoS One. 2015 Apr 20;10(4):e0123219. doi: 10.1371/journal.pone.0123219 (PMC4404324; doi:10.1371/journal.pone.0123219)
Supplement: S1 Table — (DOCX) [file pone.0123219.s003.docx]

**Table S1.** LC-MS/MS protein identifications from outer membrane preparations.

| **Uniprot ID** | **Protein** | **MW** | **pI** | **Mascot Score** | **Peptide Hits** | **% SC** | **Localization** | **Putative Function** | **Culture media & Phase of growth** |
| --- | --- | --- | --- | --- | --- | --- | --- | --- | --- |
| W8V0Y7 | IutA | 81.2 | 4.7 | 8274 | 49 | 73 | OM (10.0) | Ferric aerobactin receptor precursor | R^E^, R^S^ |
| W8UVJ1 | TolC | 65.7 | 9.3 | 7874 | 25 | 67 | OM (10) | Efflux, protein secretion | L^E^, L^S^, R^E^, R^S^ |
| W8V3K9 | OmpA | 40.7 | 6.0 | 5934 | 28 | 79 | OM (10) | AMP-resistance, serum resistance | L^E^, L^S^, R^E^, R^S^ |
| W8UTX3 | FimD | 95.8 | 6.5 | 5201 | 40 | 64 | OM (10) | Exports fimbrial subunits | L^E^, L^S^, R^E^, R^S^ |
| W8VC77 | FepA3 | 87.7 | 5.8 | 5196 | 34 | 45 | OM (10) | Ferrienterobactin receptor | L^E^, L^S^, R^E^, R^S^ |
| W8VDF6 | LptD | 88.4 | 5.1 | 5101 | 40 | 63 | OM (10) | LPS assembly | L^E^, L^S^ |
| W8UWB6 | CirA | 75.0 | 5.2 | 5040 | 50 | 78 | OM (10) | Catecholate receptor | L^E^, L^S^, R^E^, R^S^ |
| W8VBB3 | OmpK36 | 40.0 | 4.4 | 4211 | 22 | 89 | OM (10) | Antibiotic resistance | L^E^, L^S^, R^E^, R^S^ |
| W8V0Y7 | FepA1 | 81.2 | 4.7 | 3688 | 34 | 66 | OM (10) | Ferrienterobactin receptor | L^E^ |
| W8VDI7 | DnaK | 69.0 | 4.7 | 3622 | 43 | 75 | C (9.97) | Heat shock chaperone | L^S^, R^S^ |
| W8VPI0 | YfaZ | 18.6 | 6.5 | 3269 | 7 | 73 | NA | Unknown | L^E^, L^S^, R^E^, R^S^ |
| W8UXZ6 | PtsI | 63.2 | 4.6 | 3206 | 37 | 68 | C (9.97) | Phosphotransferase | R^S^ |
| W8V6I5 | HmuR | 86.6 | 5.4 | 3056 | 34 | 46 | OM (9.95) | Hemin receptor | R^E^, R^S^ |
| W8VKB3 | SlyB | 15.2 | 9.4 | 2614 | 11 | 77 | OM (9.92) | Membrane integrity | L^E^, L^S^, R^E^, R^S^ |
| W8VGP1 | BtuB | 68.1 | 5.0 | 2534 | 18 | 46 | OM (10) | Cobalamin synthesis | L^E^, L^S^ |
| W8VEP4 | OmpX | 18.7 | 9.1 | 2492 | 13 | 65 | OM (10) | Bacteriocin resistance | L^E^, L^S^, R^E^, R^S^ |
| W8VGK4 | TufA | 43.2 | 5.2 | 2481 | 33 | 91 | C (9.97) | Elongation factur Tu 1 | R^S^ |
| W8VFS0 | FhuA | 81.3 | 5.6 | 2289 | 23 | 42 | OM (10) | Ferrichrome receptor | L^E^, L^S^, R^E^, R^S^ |
| W8VFR1 | BamA | 89.9 | 5.1 | 2247 | 25 | 43 | OM (10) | Protein folding, permeability | L^E^, L^S^, R^E^, R^S^ |
| W8VE47 | GroL | 57.1 | 4.7 | 2234 | 28 | 67 | C (9.97) | Chaperonin, protein folding | L^E^, L^S^, R^E^, R^S^ |
| W8VGG5 | LamB1 | 49.8 | 4.9 | 2097 | 13 | 41 | OM (10) | Maltoporin | L^E^, L^S^, R^E^, R^S^ |
| W8VNG3 | AcrA | 44.6 | 8.4 | 2090 | 18 | 68 | CM (10) | Multidrug efflux pump | R^E^, R^S^ |
| W8V235 | YbhC | 45.5 | 7.2 | 1861 | 15 | 51 | OM (9.93) | Putative thioesterase, lipoprotein | R^E^, R^S^ |
| W8VC05 | Pal | 19.7 | 7.4 | 1784 | 10 | 71 | OM (10) | Serum-resistance, inflammation | L^S^, R^E^, R^S^ |
| W8VB44 | FadL | 47.8 | 5.0 | 1719 | 10 | 37 | OM (10) | Fatty acid transport | L^E^, L^S^, R^E^, R^S^ |
| W8VDZ7 | MipA | 28.8 | 5.2 | 1530 | 10 | 66 | OM (10) | Outer membrane integrity | L^E^, L^S^, R^E^, R^S^ |
| W8UZK8 | FoxA | 78.0 | 5.2 | 1517 | 23 | 41 | OM (10) | Ferrioxamine receptor | R^E^, R^S^ |
| W8V8D3 | Wzi | 55.7 | 5.5 | 1475 | 16 | 38 | OM (9.52) | Polysaccharide export | L^E^, L^S^, R^S^ |
| W8V0Q4 | GapA | 35.9 | 6.4 | 1421 | 22 | 78 | C (9.97) | Carbohydrate synthesis, glycolysis | R^S^ |
| W8VFG7 | AcrB | 113.2 | 5.3 | 1405 | 17 | 22 | CM (10.0) | Multidrug efflux pump | R^S^ |
| W8V7P6 | PmbA | 51.2 | 5.7 | 1378 | 17 | 46 | C (9.97) | Metalloprotease, microcin maturation | R^S^ |
| W8V0V3 | YncD | 77.0 | 5.7 | 1377 | 15 | 35 | OM (10) | TonB receptor, metal/nutrient uptake | L^E^, L^S^ |
| W8VAD7 | HmuS | 38.8 | 6.1 | 1298 | 18 | 60 | C (9.97) | Hemin transporter, methyltransferase | R^S^ |
| W8VJ12 | HldE | 51.1 | 5.0 | 1292 | 18 | 55 | C (9.97) | LPS & Nucleotide biogenesis | R^S^ |
| W8VM68 | RpsA | 61.1 | 4.7 | 1258 | 17 | 33 | C (9.97) | SSU ribosomal protein S1P | R^S^ |
| W8VQV5 | Bla2 | 31.2 | 7.9 | 1243 | 13 | 61 | P (6.58) | Beta-lactam resistance | R^E^, R^S^ |
| W8UU32 | YiaD | 22.4 | 10.1 | 1238 | 9 | 72 | OM (10) | Membrane integrity | L^S^ |
| W8VA83 | Ygau | 15.9 | 5.1 | 1209 | 12 | 78 | NA | Unknown (LysM domain) | L^E^, L^S^ |
| W8VCC2 | FepA2 | 80.4 | 5.5 | 1192 | 12 | 29 | OM (10) | Ferrienterobactin receptor | L^E^, R^S^ |
| W8VM60 | AsnS | 52.4 | 5.0 | 1155 | 18 | 38 | C (10.0) | Asparagine tRNA ligase | R^S^ |
| W8VLL9 | OmpW | 24.3 | 5.2 | 1149 | 5 | 42 | OM (10) | Unknown porin | R^S^ |
| W8VJJ2 | VacJ | 31.7 | 5.5 | 1107 | 10 | 44 | OM (4.94) | Phospholipid trafficking | L^E^, R^E^, R^S^ |
| W8V6H8 | HslU | 49.7 | 5.2 | 1095 | 16 | 36 | C (9.97) | ATP-dependent protease ATPase | R^S^ |
| W8V5J5 | HflK | 45.5 | 5.9 | 1094 | 13 | 43 | C (5.48) | Regulates FtsH, proteolysis | L^E^, L^S^, R^E^, R^S^ |
| W8USF4 | FusA | 77.5 | 5.0 | 1032 | 9 | 20 | C (9.97) | Elongation factor G | L^S^, R^S^ |
| W8VJ46 | Pgk | 41.1 | 4.9 | 1021 | 16 | 53 | C (9.97) | Carbohydrate degradation, glycolysis | R^E^ |
| W8VMV0 | AhpC | 20.7 | 4.9 | 1014 | 13 | 72 | C (9.97) | Peroxiredoxin | L^S^, R^S^ |
| W8V5N2 | HtpG | 74.2 | 5.1 | 994 | 14 | 27 | C (9.97) | Heat shock chaperone | R^S^ |
| W8UYV5 | Wza | 41.4 | 6.0 | 942 | 13 | 47 | OM (9.92) | Polysaccharide export | L^S^, R^E^ |
| W8VNV7 | MetQ | 29.3 | 5.0 | 903 | 12 | 62 | CM (9.97) | Methionine transporter permease | L^E^, R^E^ |
| W8VD34 | GloB | 28.5 | 5.9 | 899 | 12 | 43 | C (9.97) | Glyoxylase, putative beta-lactamase | R^S^ |
| W8URF3 | GlpK | 56.0 | 5.4 | 892 | 12 | 26 | C (9.97) | Glycerol Kinase, polyol metabolism | R^S^ |
| W8VF34 | ClpA | 104.4 | 5.7 | 890 | 13 | 22 | C (9.97) | ATP-dep ATPase, protease chaperone | R^S^ |
| W8V4Z1 | YloB | 98.1 | 6.0 | 880 | 12 | 17 | CM (10) | Calcium transporting ATPase | R^E^ |
| W8UVW8 | Pta | 79.0 | 5.1 | 866 | 11 | 26 | C(9.26) | Acetate metabolism | L^S^ |
| W8VEJ1 | IlvC | 58.4 | 5.5 | 859 | 14 | 32 | C (9.97) | Ketol-acid isomerase | R^E^, R^S^ |
| W8VGT9 | AtpD | 50.2 | 4.7 | 837 | 12 | 38 | C (9.12) | Proton transport, ATP synthesis | L^E^, R^S^ |
| W8VJF8 | BamB | 42.0 | 4.6 | 837 | 8 | 34 | OM (4.96) | Outer membrane assembly | L^E^, R^E^, R^S^ |
| W8UV53 | Pnp | 76.8 | 4.9 | 809 | 12 | 23 | C (9.97) | RNA processing, Rnase P | L^S^ |
| W8V1E5 | SerS | 48.6 | 5.4 | 786 | 11 | 30 | C (10.0) | Serine tRNA ligase | R^S^ |
| W8VEY8 | Aac6'b | 22.3 | 4.8 | 752 | 8 | 40 | C (9.26) | Aminoglycoside resistance | R^E^, R^S^ |
| W8USR5 | Ttg2C | 20.2 | 4.8 | 747 | 9 | 51 | NA | Toluene ABC transport permease | R^S^ |
| W8VIX0 | MreB | 39.5 | 5.2 | 740 | 12 | 41 | C(9.97) | Shape-determining protein | R^E^ |
| W8V299 | Pgm | 59.2 | 5.3 | 731 | 10 | 25 | C (8.96) | Glucose metabolism | R^E^ |
| W8VBK1 | PflB | 85.1 | 5.6 | 716 | 10 | 18 | C (9.97) | Pyruvate metabolism | L^S^ |
| W8VCC3 | Lpp | 8.4 | 9.5 | 674 | 4 | 60 | OM (9.93) | Colonization factor | L^E^, L^S^, R^E^, R^S^ |
| W8VCV3 | Tsx | 33.5 | 5.1 | 670 | 8 | 33 | OM (10) | Bacteriophage receptor | L^E^, L^S^, R^E^ |
| W8UTK7 | IlvB | 60.1 | 5.1 | 638 | 8 | 24 | C(9.97) | Amino acid biosynthesis | R^E^ |
| W8USN9 | YhcB | 15.3 | 5.3 | 628 | 6 | 53 | CM (9.82) | Unknown | R^E^, R^S^ |
| W8VJB0 | NorV | 54.9 | 4.7 | 627 | 9 | 23 | C (9.97) | Flavorubredoxin | R^S^ |
| W8VBK4 | DmsA | 91.3 | 6.6 | 625 | 8 | 15 | CM (9.98) | Dimethyl sulfoxide reductase | L^S^ |
| W8V567 | ScrY | 56.2 | 5.4 | 620 | 6 | 23 | OM (10) | Sucrose porin | L^S^ |
| W8VCT6 | YajG | 20.8 | 9.10 | 617.56 | 6 | 55 | NA | Hypothetical | R^S^ |
| W8V9G7 | FitA | 78.4 | 5.3 | 617 | 9 | 24 | OM (9.95) | Ferric copragen receptor | R^E^ |
| W8VF09 | EntC | 43.3 | 5.4 | 605 | 10 | 33 | C (9.26) | Siderophore biogenesis | R^S^ |
| W8VEQ2 | AtpF | 17.3 | 5.6 | 583 | 7 | 53 | CM (9.82) | ATP synthesis | L^E^, R^E^, R^S^ |
| W8UVI6 | BamC | 37.2 | 7.3 | 577 | 9 | 41 | OM (9.92) | Outer membrane protein assembly | L^E^, L^S^, R^E^ |
| W8VGS4 | GuaA | 51.8 | 5.2 | 572 | 9 | 32 | C (9.97) | Purine biosynthesis | R^E^ |
| W8UXP0 | AdhE | 95.8 | 6.4 | 571 | 8 | 13 | C (9.26) | Alcohol metabolism | L^S^ |
| W8VD21 | PepD | 53.5 | 5.2 | 569 | 9 | 23 | C (8.96) | Serine protease Peptidase D | R^S^ |
| W8VPS6 | YtfM | 64.6 | 9.4 | 560 | 8 | 18 | OM (10) | Autotransporter assembly | L^S^ |
| W8UY75 | AckA | 43.2 | 5.9 | 551 | 8 | 29 | C (9.97) | Acetate kinase | R^S^ |
| W8VIX8 | Yhbg | 26.8 | 5.6 | 544 | 8 | 45 | C (9.12) | LPS export | R^E^ |
| W8VC36 | YbeZ | 39.2 | 5.7 | 530 | 8 | 30 | C (9.97) | ATP-binding cytoplasmic protein | R^S^ |
| W8VQC9 | CobA | 43.2 | 4.5 | 519 | 8 | 31 | CM (9.82) | Uroporphyrinogen methylase | R^E^, R^S^ |
| W8V0M6 | TyrS | 49.0 | 5.6 | 511 | 7 | 26 | C (9.97) | Tyrosine tRNA ligase | R^S^ |
| W8V8B7 | Kbl | 43.1 | 5.6 | 497 | 8 | 31 | C (9.97) | Glycine synthesis | R^S^ |
| W8UXK1 | HcsA | 65.4 | 4.8 | 494 | 7 | 14 | C (9.97) | Polysaccharide biosynthesis | R^S^ |
| W8VBT5 | YedD | 15.8 | 8.5 | 489 | 6 | 56 | NA | Unknown | R^E^, R^S^ |
| W8V3G4 | FklB | 69.5 | 5.0 | 467 | 7 | 13 | C (9.97) | Isomerase, protein folding | R^S^ |
| W8V7W1 | Ccl | 59.2 | 9.6 | 462 | 8 | 22 | P (9.83) | Cloacin, hydrolyzes 16S RNA | R^E^ |
| W8V340 | CysS | 54.0 | 5.1 | 460 | 5 | 14 | C (9.97) | Cysteine-tRNA ligase | R^S^ |
| W8V1U9 | M32 Peptidase | 56.4 | 5.2 | 459 | 6 | 18 | C (8.96) | Uncharacterized Metallo-peptidase | R^S^ |
| W8UYP1 | YohG | 52.2 | 5.6 | 436 | 7 | 17 | OM (10.0) | Putative multidrug resistance efflux | R^S^ |
| W8UUK2 | OmpR | 29.2 | 6.0 | 430 | 8 | 39 | C (9.97) | OMP protein txn, response regulator | R^S^ |
| W8V5X1 | FimF | 22.1 | 9.9 | 424 | 5 | 36 | EX (9.72) | Fimbrial length regulation | L^E^, L^S^ |
| W8V6G2 | Ppc | 99.9 | 5.8 | 421 | 6 | 11 | C (8.96) | Phosphoenolpyruvate carboxylase | R^S^ |
| W8VB78 | YfbQ | 46.6 | 6.2 | 421 | 6 | 20 | C (9.97) | Aminotransferase | R^S^ |
| W8VJB3 | RecA | 37.9 | 4.9 | 410 | 6 | 24 | C(9.97) | Recombination | R^E^ |
| W8VBX5 | S41 peptidase | 78.3 | 6.4 | 405 | 7 | 14 | CM (10) | Serine peptidase | L^S^ |
| W8UXV5 | SufS | 43.7 | 5.7 | 379 | 5 | 20 | C (9.97) | Cysteine desulfurase | R^S^ |
| W8VDC0 | FtsZ | 40.3 | 4.5 | 376 | 6 | 24 | C (9.12) | Z-ring component for cell division | R^S^ |
| W8VNJ0 | Lon | 91.4 | 5.7 | 376 | 6 | 10 | C (9.97) | Protease | R^E^ |
| W8VMN6 | GpmA | 28.3 | 5.6 | 371 | 6 | 28 | C (8.96) | Phosphoglycerate mutase, glycolysis | R^S^ |
| W8VBM4 | Ybjp | 18.5 | 6.9 | 370 | 4 | 47 | NA | Unknown | L^E^, L^S^, R^S^ |
| W8V9Y3 | OprD | 49.6 | 4.8 | 369 | 4 | 14 | OM (10) | Amino acid transporter, protease | L^E^, L^S^ |
| W8VFI3 | ClpX | 48.3 | 6.1 | 364 | 5 | 17 | C (9.97) | ATP-dep ATPase, protease chaperone | R^S^ |
| W8VD90 | Hpt | 20.5 | 4.9 | 357 | 5 | 50 | C (9.97) | Hypoxanthine transferase | R^S^ |
| W8V5J1 | PurA | 47.1 | 5.2 | 357 | 5 | 17 | C(9.97) | Purine biosynthesis | R^E^ |
| W8V2D5 | RlpA | 39.1 | 5.4 | 357 | 5 | 13 | EX (9.65) | Unknown, lipoprotein | R^E^ |
| W8VKY5 | YdfH | 26.5 | 5.9 | 354 | 5 | 35 | C (9.97) | Putative DNA binding activity | R^S^ |
| W8VBT9 | CcmA | 63.2 | 4.8 | 353 | 5 | 13 | C (9.97) | Cytochrome c biogenesis | R^S^ |
| W8V807 | YfaZ2 | 18.6 | 4.7 | 347 | 3 | 21 | NA | Unknown | L^E^, L^S^ |
| W8VGZ2 | Sul | 28.4 | 5.9 | 344 | 5 | 40 | C (9.26) | Sulfonamide resistance protein | R^S^ |
| W8V5L4 | Efp | 20.6 | 4.8 | 330 | 5 | 36 | C (9.97) | Elongation factor P | R^S^ |
| W8VCW8 | CheY | 30.9 | 5.6 | 328 | 6 | 32 | C (9.97) | Putative CheY-like txnal regulator | R^S^ |
| W8V687 | TatB | 19.1 | 4.9 | 327 | 5 | 37 | CM (9.82) | Sec-dependent translocase | R^E^ |
| W8VLH4 | FabL | 27.9 | 5.3 | 326 | 5 | 25 | CM (10) | Enoyl reductase | R^E^ |
| W8V4S6 | ThrC | 46.6 | 5.4 | 324 | 5 | 15 | C (9.97) | Threonine synthase | R^S^ |
| W8V0J8 | Fiu | 82.9 | 5.6 | 323 | 5 | 10 | OM (10) | Siderophore receptor | L^S^ |
| W8V404 | ProS | 63.5 | 4.9 | 321 | 4 | 10 | C (10.0) | Proline-tRNA ligase | R^S^ |
| W8V265 | TolB | 46.0 | 8.8 | 321 | 6 | 15 | P (10) | Protein transport | R^E^ |
| W8UZH6 | ManX | 34.8 | 5.9 | 320 | 4 | 16 | C (9.97) | Mannose transporter component | R^E^, R^S^ |
| W8UW71 | FimC | 26.2 | 10.0 | 319 | 5 | 25 | P (10) | Fimbrial chaperone | L^E^ |
| W8V2V3 | TrpE | 57.4 | 5.2 | 301 | 5 | 13 | C (9.97) | Anthranilate synthase | R^S^ |
| W8V7V3 | PurF | 56.5 | 5.3 | 298 | 5 | 15 | C(9.97) | Purine biosynthesis | R^E^ |
| W8VMR8 | ChiP | 52.5 | 5.0 | 294 | 4 | 12 | NA | Chitobiotin porin | L^E^ |
| W8VD59 | DapD | 29.8 | 5.3 | 288 | 4 | 15 | C(9.97) | Amino acid biosynthesis | R^E^ |
| W8V8N8 | BipA | 67.2 | 5.0 | 287 | 6 | 16 | CM (7.88) | GTPase | R^S^ |
| W8V499 | AceF | 65.9 | 5.0 | 279 | 4 | 13 | C (9.97) | Pyruvate dehydrogenase, glycolysis | L^S^ |
| W8V9F3 | PykF | 50.6 | 5.5 | 278 | 4 | 13 | C (9.97) | Pyruvate kinase I | R^S^ |
| W8VA20 | FimA | 24.8 | 9.0 | 276 | 2 | 18 | EX (9.72) | Fimbrial adhesion | L^S^ |
| W8VMT4 | LptE | 21.4 | 7.4 | 269 | 3 | 19 | OM (9.92) | LPS assembly | L^E^, L^S^ |
| W8UTT1 | TpiA | 26.9 | 5.7 | 268 | 4 | 17 | C (9.97) | Isomerase, gluconeogenesis | R^S^ |
| W8VH91 | Exc2 | 15.6 | 9.7 | 264 | 3 | 43 | NA | Entry exclusion protein | L^E^, R^E^ |
| W8UYC1 | FrmA | 39.2 | 5.8 | 254 | 4 | 19 | C (9.97) | Class III alcohol dehydrogenase | R^S^ |
| W8VCV6 | YajC | 13.0 | 9.9 | 253 | 3 | 25 | CM (10) | Component of Sec translocase | R^E^, R^S^ |
| W8VGA7 | RplL | 15.8 | 6.2 | 245 | 3 | 23 | C 9(.97) | 50s ribosomal protein L7/L12 | R^E^ |
| W8V3C1 | LamB2 | 50.4 | 4.9 | 242 | 3 | 10 | OM (9.93) | Maltoporin | L^S^ |
| W8V3Z0 | LysR | 33.2 | 7.1 | 239 | 1 | 12 | C(9.97) | Transcriptional regulator | R^S^ |
| W8UTH7 | GyrB | 90.1 | 5.7 | 232 | 4 | 10 | C (9.97) | DNA gyrase B | R^S^ |
| W8VKY8 | YnfB | 13.0 | 8.9 | 232 | 3 | 34 | NA | Unknown | R^E^, R^S^ |
| W8VGC9 | EcnB | 4.8 | 8.9 | 221 | 1 | 40 | CM (9.97) | Bacteriolysin antidote | L^E^, L^S^ |
| W8VL81 | MocA | 38.8 | 5.8 | 214 | 4 | 24 | C (8.96) | Oxioreductase | R^S^ |
| W8VJ58 | FimH | 31.6 | 5.8 | 208 | 2 | 16 | CM (4.65) | Fimbrial length regulation | L^S^ |
| W8VBE2 | RsuA | 26.6 | 5.9 | 204 | 3 | 29 | C (9.97) | Pseudouridine synthase | R^S^ |
| W8UX60 | MinD | 29.6 | 5.0 | 203 | 3 | 16 | C (9.12) | Septum site determining protein | R^S^ |
| W8UW20 | Epd | 37.4 | 6.0 | 196 | 4 | 20 | C (9.97) | Erythrose-phosphate dehydrogenase | R^S^ |
| W8V4I4 | SdhB | 26.7 | 6.1 | 196 | 3 | 17 | CM (9.82) | Succinate dehydrogenase | L^E^ |
| W8VNW0 | CutF | 26.4 | 5.8 | 194 | 3 | 13 | OM (9.93) | Copper efflux | R^E^ |
| W8VE23 | HflC | 37.6 | 7.6 | 192 | 3 | 11 | C (8.96) | Regulator of lysogenization and FtsH | R^E^, R^S^ |
| W8V016 | GalU | 32.6 | 5.0 | 191 | 3 | 11 | C(9.97) | Glucose metabolism | R^E^ |
| W8V1R4 | PdxA | 35.1 | 6.0 | 191 | 2 | 16 | C (9.97) | Pyridoxal phosphate biosynthetic | R^S^ |
| W8VJ59 | FimI | 22.6 | 9.9 | 190 | 2 | 10 | Ex (9.71) | Fimbrial protein | R^E^, R^S^ |
| W8V0X0 | YcfD | 42.6 | 4.6 | 189 | 3 | 14 | C (9.97) | Hypothetical cytosolic enzyme | R^S^ |
| W8UZE1 | FklB | 24.0 | 5.5 | 185 | 3 | 15 | C (9.97) | Isomerase, protein folding | R^E^ |
| W8VG23 | YjjG | 25.2 | 4.5 | 183 | 2 | 16 | C (9.26) | Hydrolase/Nucleotidase | R^S^ |
| W8UTX0 | WalW | 39.9 | 6.0 | 182 | 4 | 11 | C (8.96) | Unknown | R^E^ |
| W8VBF3 | PfkA | 33.8 | 5.1 | 179 | 4 | 15 | C (9.26) | Pyruvate metabolism | R^S^ |
| W8V8V1 | HtpX | 32.0 | 6.3 | 178 | 2 | 10 | CM (10.0) | Membrane protease | R^S^ |
| W8V7S9 | RpsF | 15.1 | 5.1 | 177 | 3 | 27 | C (9.97) | 30S ribosomal protein S6 | R^S^ |
| W8VCN3 | TehB | 22.0 | 5.1 | 175 | 3 | 14 | NA | Tellurite resistance methyltransferase | R^S^ |
| W8VEB2 | PyrD | 37.7 | 7.8 | 173 | 3 | 11 | CM (7.88) | Dihydroorotate dehydrogenase | R^S^ |
| W8V912 | RplE | 20.3 | 9.9 | 170 | 3 | 26 | C (9.97) | 50s ribosomal protein L5 | L^S^ |
| P0A7V0 | RpsB | 26.7 | 6.7 | 167 | 2 | 12 | C 9(.97) | 30S ribosomal protein S2 | R^E^ |
| W8V428 | Tsf | 33.7 | 5.2 | 165 | 3 | 13 | C (9.97) | Elongation factor Ts | R^S^ |
| W8V6G4 | FhuD | 33.0 | 7.4 | 163 | 3 | 12 | P (10.0) | Fe (3+)-hydroxamate transporter | R^S^ |
| W8VFQ2 | RcsF | 14.3 | 9.5 | 159 | 2 | 21 | OM (9.92) | Signal transduction | L^S^ |
| W8V916 | RpsE | 17.6 | 10.7 | 154 | 2 | 21 | C (9.97) | 30S ribosomal protein S5 | L^E^ |
| W8UZI7 | Slp | 23.4 | 10.2 | 153 | 2 | 14 | OM (9.92) | Membrane stability | L^S^ |
| W8URS1 | YiaF | 27.1 | 8.9 | 151 | 3 | 12 | OM (9.82) | Unknown | R^E^ |
| W8V6E1 | Eno | 47.3 | 4.9 | 150 | 2 | 12 | C (9.97) | Glycolysis, carbohydrate degradation | L^E^ |
| W8VBS9 | Hydrolase | 26.4 | 5.8 | 150 | 3 | 9 | NA | Hydrolase | R^S^ |
| W8VF57 | Bar | 19.7 | 7.8 | 146 | 3 | 13 | NA | Phosphinothricin N-acetyltransferase | R^S^ |
| W8VLN3 | OsmE | 12.3 | 8.5 | 142 | 3 | 36 | NA | Osmotic-stress inducible protein | R^E^, R^S^ |
| W8V038 | YdiY | 27.3 | 4.9 | 141 | 3 | 10 | OM (9.52) | Unknown | L^S^, R^S^ |
| W8V4S5 | MurA | 44.5 | 5.5 | 140 | 2 | 14 | C(9.97) | Peptidoglycan biosynthesis | R^E^ |
| W8VJH4 | AhpC2 | 17.6 | 4.8 | 137 | 2 | 18 | C (9.97) | Peroxiredoxin | R^S^ |
| W8VD84 | UspA | 16.2 | 9.5 | 137 | 2 | 29 | C (8.96) | Stress-induced protein | R^E^ |
| A6T5Q5 | OmpU | 43.2 | 5.2 | 135 | 3 | 12 | NA | Hypothetical | L^S^ |
| W8VAA4 | Syd | 20.3 | 5.0 | 135 | 2 | 24 | CM (9.82) | Putative Sec regulator | R^S^ |
| W8V103 | FabF | 42.8 | 5.4 | 133 | 2 | 12 | CM (9.82) | 3-oxoacyl-synthase II | R^S^ |
| W8VED4 | RplA | 24.7 | 10.1 | 125 | 2 | 14 | C (9.97) | 50s ribosomal protein L1 | L^E^ |
| W8V2N3 | NfnB | 24.0 | 5.6 | 119 | 2 | 10 | C(9.97) | Nitroreductase | R^S^ |
| W8VLH4 | FabI | 27.9 | 5.3 | 118 | 2 | 14 | CM (10) | Enoyl reductase | R^S^ |
| W8V6Q7 | AtpH | 19.5 | 4.8 | 115 | 2 | 18 | C (9.97) | ATP-synthase, delta chain | R^S^ |
| W8US41 | DcrB | 19.7 | 4.9 | 106 | 2 | 14 | P (9.44) | Aids in phage adsorption | L^S^ |
| W8VDP4 | Rha | 27.6 | 6.2 | 101 | 2 | 12 | C (8.96) | Txnal regulator, phage anti-repressor | R^S^ |
| W8VH18 | Hsp20 | 21.5 | 5.2 | 100 | 2 | 11 | NA | Heat shock chaperone | R^S^ |
| W8V929 | TsaC | 22.4 | 5.8 | 99 | 2 | 21 | C (8.96) | TC-AMP synthesis | R^S^ |
| W8UVM4 | YfeX | 33.1 | 5.1 | 98 | 2 | 11 | C (8.96) | Deferrochetalase | R^S^ |
| W8V3U3 | AmpD | 31.2 | 6.6 | 96 | 2 | 12 | P (4.99) | AMP-deaminase | R^S^ |
| W8VIX8 | LptB | 26.8 | 5.6 | 88 | 2 | 17 | C (9.12) | LPS assembly | R^S^ |

Proteins from outer membrane preparations were identified by liquid chromatography combined with tandem mass spectrometry. The number of peptide hits was reduced to fit the stringent Paris Guidelines. The putative function for each protein was taken from annotations in the Uniprot database or when referenced from individual studies. The cellular localization of each protein was predicted using the software Psortb 3.0, which assigns a confidence estimate between 0 and 10 for each cellular location: OM, outer membrane; CM, cytoplasmic membrane; C, cytoplasm; EX, extracellular; P, periplasmic; NA, unknown. Each spot number refers to a sample extracted from a polyacrylamide two-dimensional gel for LC-MS/MS analysis. Other abbreviations: L^E^, LB & mid-exponential phase of growth; L^S^, LB & stationary phase of growth; R^E^, RPMI & mid-exponential phase of growth; R^S^, RPMI & stationary phase of growth; ATP, adenosine triphosphate.
