# Supplementary material for: Identification of Outer Membrane and Exoproteins of Carbapenem-Resistant Multilocus Sequence Type 258 Klebsiella pneumoniae
Source: PLoS One. 2015 Apr 20;10(4):e0123219. doi: 10.1371/journal.pone.0123219 (PMC4404324; doi:10.1371/journal.pone.0123219)
Supplement: S2 Table — (DOCX) [file pone.0123219.s004.docx]

**Table S2.** LC-MS/MS protein identifications from spent culture media.

| **Uniprot ID** | **Protein** | **MW** | **pI** | **Mascot Score** | **Peptide Hits** | **% SC** | **Psortb Localization** | **Putative Function** | **Culture media** |
| --- | --- | --- | --- | --- | --- | --- | --- | --- | --- |
| W8USF4 | FusA | 77.5 | 5.0 | 3723 | 33 | 61 | C (9.97) | Translation elongation factor | TSB |
| W8VE47 | GroL | 57.1 | 4.7 | 3722 | 36 | 81 | C (9.97) | Chaperonin, protein folding | M63, TSB |
| W8VBB3 | OmpK36 | 40.0 | 4.4 | 3269 | 24 | 80 | OM (10) | Antibiotic resistance, activates complement | M63, TSB |
| W8V3K9 | OmpA | 40.7 | 6.0 | 3071 | 23 | 78 | OM (10) | Immune evasion, AMP resistance | M63, TSB |
| W8V6E1 | Eno | 47.3 | 4.9 | 2789 | 24 | 73 | C (9.97) | Enoyl reductase | TSB |
| W8VDI7 | DnaK | 69.0 | 4.7 | 2599 | 28 | 55 | C (9.97) | Osmotic-shock chaperone | M63, TSB |
| W8V7W1 | Ccl | 59.2 | 9.6 | 2476 | 21 | 45 | P (9.83) | Cloacin, hydrolyzes 16S RNA | M63, TSB |
| W8VGK4 | TufA | 43.2 | 5.2 | 2198 | 19 | 57 | C (9.97) | Translation elongation factor | M63, TSB |
| W8V9F3 | PykF | 50.6 | 5.5 | 1869 | 20 | 51 | C (9.97) | Pyruvate metabolism | TSB |
| W8UV53 | Pnp | 76.8 | 4.9 | 1828 | 26 | 52 | C (9.97) | RNA processing, Rnase P | TSB |
| W8V3G9 | Tig | 48.1 | 4.7 | 1776 | 22 | 58 | C (8.96) | Protein folding and export | TSB |
| W8VED0 | RpoC | 155.1 | 6.5 | 1773 | 26 | 24 | C (9.97) | DNA-directed RNA polymerase, subunit B' | TSB |
| W8VGT9 | AtpD | 50.2 | 4.7 | 1660 | 21 | 75 | C (9.12) | Coupled proton transport, ATP synthesis | TSB |
| W8V8R9 | AtpA | 55.1 | 5.6 | 1555 | 19 | 49 | C (9.97) | Proton transport, ATP synthesis | TSB |
| W8VJ46 | Pgk | 41.1 | 4.9 | 1409 | 19 | 59 | C (9.97) | Carbohydrate degradation, glycolysis | M63, TSB |
| W8VBK1 | PflB | 85.1 | 5.6 | 1366 | 20 | 32 | C (9.97) | Pyruvate fermentation | TSB |
| W8V0Q4 | GapA | 35.9 | 6.4 | 1185 | 14 | 60 | C (9.97) | Carbohydrate degradation, glycolysis | M63, TSB |
| W8V765 | OsmY | 21.3 | 7.9 | 1177 | 10 | 54 | P (9.76) | Induced by osmotic pressure | TSB |
| W8V7P0 | CysK | 34.5 | 5.8 | 1163 | 14 | 69 | C (9.97) | Cysteine synthase | M63, TSB |
| W8VJF9 | GuaB | 54.5 | 6.4 | 1101 | 13 | 43 | C (9.97) | Guanine synthesis | TSB |
| W8VEP4 | OmpX | 18.7 | 9.1 | 1100 | 8 | 53 | OM (10) | Bacteriocin resistance | M63, TSB |
| W8UVW8 | Pta | 79.0 | 5.1 | 1066 | 15 | 32 | C(9.26) | Acetate metabolism | TSB |
| W8UW84 | MrkA | 20.9 | 5.1 | 1058 | 7 | 36 | Ex (10) | Type 4 fimbrial subunit | M63, TSB |
| W8V299 | Pgm | 59.2 | 5.3 | 1028 | 14 | 33 | C (8.96) | Glucose metabolism | TSB |
| W8VIT1 | LivJ | 39.4 | 6.5 | 1017 | 12 | 55 | P (10.0) | Leu/Ile/Val-binding protein | M63, TSB |
| W8VM68 | RpsA | 61.1 | 4.7 | 996 | 12 | 26 | C (9.97) | SSU ribosomal protein S1P | TSB |
| W8UXP0 | AdhE | 95.8 | 6.4 | 993 | 14 | 24 | C (9.26) | Hydrogen peroxide resistance | TSB |
| W8V499 | AceF | 65.9 | 5.0 | 973 | 14 | 33 | C (9.97) | Acetyl-CoA formation | TSB |
| W8V618 | RpoB | 151.7 | 5.1 | 970 | 16 | 16 | C (9.97) | DNA-directed RNA polymerase, subunit B | TSB |
| W8VAR7 | ClpB | 95.7 | 5.3 | 951 | 12 | 19 | C (9.97) | Protein unfolding during stress response | TSB |
| W8V727 | TalB | 35.2 | 4.8 | 937 | 14 | 56 | NA | Carbohydrate degradation | M63, TSB |
| W8VPR7 | TrxC | 44.4 | 6.2 | 921 | 11 | 33 | NA | Thioredoxin-like protein | TSB |
| W8UTR9 | TktB | 76.4 | 6.0 | 906 | 14 | 28 | C (9.26) | Transketolase | TSB |
| W8VJF1 | GlyA | 45.7 | 6.2 | 905 | 14 | 37 | C (9.97) | Amino acid biosynthesis | TSB |
| W8V282 | GltA | 48.0 | 6.2 | 846 | 12 | 39 | C (9.97) | Citrate synthesis | TSB |
| W8VQV5 | KPC-8 | 31.2 | 7.9 | 837 | 8 | 41 | P (6.58) | Carbapenemase | TSB |
| W8VPP2 | ValS | 108.2 | 5.1 | 835 | 16 | 26 | C (9.97) | Valine-tRNA ligase | TSB |
| W8VDK8 | Hypothetical | 110.7 | 5.0 | 829 | 10 | 15 | NA | Unknown | TSB |
| W8VDA3 | LpdA | 50.6 | 5.8 | 798 | 12 | 32 | C (9.97) | Glycine cleavage, glycolysis | TSB |
| W8VB34 | KatE | 83.4 | 5.3 | 773 | 13 | 24 | C (9.97) | Hydrogen peroxide resistance | TSB |
| W8VJT7 | AspS | 66.4 | 5.3 | 772 | 11 | 28 | C (10.0) | Aspartate-tRNA synthetase | TSB |
| W8V9Y6 | FbaA | 39.1 | 5.6 | 751 | 10 | 37 | C (9.97) | Glycolysis | TSB |
| W8V8C3 | GpmI | 59.0 | 5.2 | 749 | 10 | 30 | C (9.97) | Phosphoglycerate | TSB |
| W8UVJ1 | TolC | 65.7 | 9.3 | 738 | 10 | 22 | OM (10) | Efflux, protein secretion | M63, TSB |
| W8V235 | YbhC | 45.5 | 7.2 | 734 | 8 | 20 | OM (9.93) | Unknown lipoprotein | M63, TSB |
| W8UWC0 | MglB | 36.9 | 6.7 | 731 | 8 | 35 | P (10.0) | Galactose transport | TSB |
| W8V265 | TolB | 46.0 | 8.8 | 717 | 9 | 30 | P (10.0) | Colicin receptor | M63, TSB |
| W8V428 | Tsf | 33.7 | 5.2 | 715 | 11 | 37 | C (9.97) | Translation elongation factor | TSB |
| W8UY75 | AckA | 43.2 | 5.9 | 713 | 11 | 43 | C (9.97) | Acetate kinase | TSB |
| W8VJL5 | Eco | 18.0 | 7.9 | 691 | 8 | 49 | P (10) | Ecotin, Serine protease inhibitor | TSB |
| W8V5N2 | HtpG | 74.2 | 5.1 | 689 | 10 | 23 | C (9.97) | Heat shock chaperone | TSB |
| W8V5S6 | UvrA | 103.9 | 6.2 | 684 | 9 | 13 | C (9.97) | DNA repair | TSB |
| W8V5Y8 | PurH | 58.2 | 5.6 | 664 | 8 | 19 | C (9.97) | Purine biosynthesis | TSB |
| W8V554 | DhaD | 40.1 | 5.4 | 655 | 8 | 31 | C (9.26) | Glycerol fermentation | TSB |
| W8UUR9 | RpoA | 36.4 | 4.8 | 651 | 9 | 38 | C (9.97) | DNA-directed RNA polymerase, subunit A | TSB |
| W8VC57 | PrsA | 36.6 | 5.6 | 635 | 7 | 28 | C (9.97) | Protein folding and export | TSB |
| W8V1E5 | SerS | 48.6 | 5.4 | 629 | 9 | 23 | C (10.0) | Serine-tRNA ligase | TSB |
| W8VLC6 | Hypothetical | 40.6 | 4.5 | 619 | 9 | 38 | NA | Phage-related protein | TSB |
| W8V8E0 | Gnd | 51.4 | 4.8 | 609 | 9 | 26 | C (8.96) | Carbohydrate degradation | TSB |
| W8V912 | RplE | 20.3 | 9.9 | 609 | 8 | 50 | C (9.97) | 50S ribosomal protein | M63 |
| W8V7A1 | PepB | 47.4 | 5.9 | 607 | 7 | 30 | C (9.97) | Intracellular peptide degradation | TSB |
| W8VDM3 | Hypothetical | 34.4 | 4.9 | 606 | 7 | 33 | NA | Putative phage protein | TSB |
| W8V4I1 | SurA | 47.0 | 6.5 | 593 | 8 | 28 | P (10.0) | Outer membrane folding and assembly | TSB |
| W8VJJ5 | FabB | 42.4 | 5.2 | 584 | 8 | 34 | C (9.97) | Fatty acid elongation | M63, TSB |
| W8VG46 | GabD | 49.5 | 5.8 | 573 | 8 | 21 | C (9.97) | Amino acid degradation | TSB |
| W8VDM5 | DeoB | 44.7 | 5.0 | 558 | 9 | 25 | C (9.97) | Pentose metabolism | TSB |
| W8VGU0 | GlmS | 66.8 | 5.6 | 554 | 9 | 21 | C (9.97) | Hexosamine synthesis | TSB |
| W8VDG4 | CarB | 117.9 | 5.0 | 552 | 9 | 12 | P (4.99) | Lysine decarboxylase | TSB |
| W8VGA7 | RplL | 15.8 | 6.2 | 540 | 6 | 52 | C (9.97) | 50S ribosomal protein | M63 |
| W8V4Q0 | AhpF | 56.0 | 5.3 | 532 | 8 | 23 | CM (7.88) | Hydrogen peroxide resistance | TSB |
| W8V5V3 | MalE | 43.2 | 8.6 | 515 | 6 | 19 | P (10.0) | Maltose transport | TSB |
| W8V7V3 | PurF | 56.5 | 5.3 | 509 | 8 | 18 | C (9.97) | Purine metabolism | TSB |
| W8VLL9 | OmpW | 24.3 | 5.2 | 508 | 3 | 21 | OM (10) | Unknown membrane protein | M63, TSB |
| W8V2V5 | CadA | 81.5 | 5.7 | 502 | 7 | 15 | C (9.97) | pH homeostasis | TSB |
| W8VB04 | TktA | 73.6 | 5.7 | 499 | 7 | 13 | C (9.26) | Transketolase | TSB |
| W8VGG5 | LamB1 | 49.8 | 4.9 | 494 | 5 | 15 | OM (10) | Maltoporin | M63 |
| W8V4P2 | DegP | 47.2 | 6.2 | 491 | 8 | 24 | P (10.0) | Periplasmic protease | TSB |
| W8VC77 | FepA3 | 87.7 | 5.8 | 491 | 5 | 12 | OM (10) | Ferrienterobactin receptor | M63 |
| W8VEV2 | SucC | 41.5 | 5.2 | 481 | 7 | 25 | C (8.96) | ATP synthesis during citric acid cycle | TSB |
| W8V1F2 | TrxB | 35.6 | 5.2 | 480 | 6 | 31 | P (4.99) | Destroys superoxide radials | TSB |
| W8V0W0 | IcdA | 47.6 | 5.4 | 477 | 8 | 23 | C (9.97) | Glyoxylate metabolism | TSB |
| W8V848 | Pgi | 61.5 | 5.8 | 477 | 7 | 20 | C (9.97) | Carbohydrate degradation, glycolysis | TSB |
| W8V340 | CysS | 54.0 | 5.1 | 473 | 7 | 23 | C (9.97) | Cysteine-tRNA ligase | TSB |
| W8V8B7 | Kbl | 43.1 | 5.6 | 469 | 7 | 21 | C (9.97) | Glycine synthesis, amino acid degradation | TSB |
| W8UUR0 | RplF | 18.8 | 10.1 | 469 | 6 | 38 | C (9.26) | 50S ribosomal protein | M63 |
| W8V830 | GlvA | 49.2 | 5.6 | 468 | 7 | 23 | C (8.96) | Carbohydrate mteabolism | TSB |
| W8VJN2 | FbaB | 39.8 | 6.4 | 462 | 5 | 18 | NA | Iron uptake | TSB |
| W8V103 | FabF | 42.8 | 5.4 | 460 | 6 | 24 | CM (9.82) | Fatty acid elongation | TSB |
| W8V5M9 | GroS | 10.3 | 5.2 | 460 | 4 | 54 | C (9.97) | Chaperonin, protein folding | M63, TSB |
| W8V2D5 | RlpA | 39.1 | 5.4 | 456 | 7 | 23 | OM (9.65) | Unknown lipoprotein | M63, TSB |
| W8V4U1 | SlmT | 74.1 | 7.4 | 426 | 7 | 13 | P (10.0) | Soluble lytic murein transglycosylase | TSB |
| W8V9A8 | OppA | 61.3 | 6.1 | 420 | 7 | 20 | P (10.0) | Oligopeptide transport | TSB |
| W8UUG9 | NlpD | 39.3 | 10.0 | 416 | 6 | 21 | OM (8.86) | M23 Endopeptidase | M63, TSB |
| W8V8W3 | PckA | 59.5 | 5.3 | 405 | 5 | 14 | C (9.97) | Carbohydrate biosynthesis | TSB |
| W8V7P6 | PmbA | 51.2 | 5.7 | 400 | 7 | 16 | C (9.97) | Metalloprotease, microcin maturation | TSB |
| W8UVB2 | UxaC | 53.7 | 5.5 | 391 | 6 | 13 | C (8.96) | Carbohydrate mteabolism | TSB |
| W8V3U4 | ProA | 44.8 | 5.4 | 384 | 6 | 14 | C (9.97) | Proline biosynthesis | TSB |
| W8V7B8 | RecT | 37.1 | 5.4 | 380 | 6 | 23 | C (8.96) | Recombination and DNA repair | TSB |
| W8V792 | SuhB | 29.3 | 6.8 | 371 | 5 | 23 | C (9.97) | Myo-inositol formation | TSB |
| W8V9C5 | PheT | 86.8 | 4.9 | 368 | 6 | 10 | C (9.97) | Protein synthesis | TSB |
| W8VJM8 | BglX | 83.3 | 5.7 | 363 | 6 | 12 | P (10) | Carbohydrate degradation | TSB |
| W8V0M6 | TyrS | 49.0 | 5.6 | 360 | 5 | 16 | C (9.97) | Tyrosine-tRNA ligase | TSB |
| W8VEX5 | Gltl | 36.1 | 8.1 | 358 | 6 | 19 | P (10.0) | Murein cleavage during cell division | TSB |
| W8VGS4 | GlnA | 51.8 | 5.2 | 353 | 6 | 23 | C (9.97) | Glutamine synthetase | TSB |
| W8V1B6 | AspC | 43.5 | 5.2 | 347 | 5 | 17 | C (9.97) | Glutamate synthesis | M63, TSB |
| W8V201 | YdgH | 33.9 | 9.6 | 339 | 4 | 18 | NA | Unknown membrane protein | TSB |
| W8VIS2 | Gor | 48.8 | 5.8 | 334 | 4 | 18 | C (9.97) | Glutathione metabolism | TSB |
| W8UU44 | DppA | 61.5 | 6.8 | 332 | 4 | 9 | P (9.76) | Dipeptide transport | M63, TSB |
| W8V378 | PepT | 46.2 | 5.3 | 331 | 5 | 21 | C (9.97) | Protease, cleaves tripeptides | TSB |
| W8VD21 | PepD | 53.5 | 5.2 | 329 | 6 | 14 | C (8.96) | Serine protease Peptidase D | TSB |
| W8VGP1 | BtuB | 68.1 | 5.0 | 324 | 5 | 13 | OM (10) | Vitamin B12 transport | M63 |
| W8V9T2 | YdcS | 42.2 | 7.6 | 321 | 3 | 12 | P (9.44) | Solute binding and transport | TSB |
| W8UUG3 | LivK | 42.7 | 8.2 | 313 | 4 | 17 | P (10.0) | Leucine transport | M63, TSB |
| W8VQ78 | RplJ | 17.8 | 9.5 | 309 | 4 | 32 | C (9.97) | 50S ribosomal protein | TSB |
| W8V7P8 | GltX | 53.5 | 5.5 | 304 | 4 | 11 | C (9.97) | Glutamate-tRNA ligase | TSB |
| W8V3D3 | MdoG | 59.0 | 9.0 | 289 | 4 | 13 | P (10) | Beta-glucan biosynthesis | TSB |
| W8V8C1 | PepQ | 50.1 | 5.7 | 289 | 4 | 12 | C (9.97) | Dipeptidase | TSB |
| W8V2L1 | DppB | 57.1 | 7.0 | 287 | 5 | 16 | P (9.76) | Dipeptide transport | TSB |
| W8VJ49 | GcvT | 41.0 | 4.9 | 287 | 4 | 15 | C (9.26) | Glycine degradation | TSB |
| W8VC14 | SucB | 44.2 | 5.6 | 282 | 5 | 14 | C (9.97) | Succinyl CoA formation,TCA cycle | TSB |
| W8V208 | ManA | 43.2 | 5.2 | 280 | 5 | 19 | C (8.96) | Mannose metabolism | TSB |
| W8VM60 | AsnS | 52.4 | 5.0 | 279 | 5 | 13 | C (10.0) | Asparagine-tRNA ligase | TSB |
| W8V0X5 | PotD | 38.8 | 5.4 | 278 | 4 | 15 | P (10.0) | Putrescine transport | TSB |
| W8URF3 | GlpK | 56.0 | 5.4 | 275 | 4 | 10 | C (9.97) | Glycerol uptake and metabolism | M63 |
| W8V751 | Yjjk | 62.2 | 5.4 | 275 | 4 | 10 | C (9.97) | ATP synthesis, electron transport | TSB |
| W8UW02 | MetK | 41.9 | 5.0 | 274 | 4 | 13 | C (9.97) | SAM formation | TSB |
| W8V649 | RplK | 14.8 | 10.1 | 268 | 4 | 33 | C (9.26) | 50S ribosomal protein | M63, TSB |
| W8VED4 | RplA | 24.7 | 10.1 | 259 | 4 | 29 | C (9.97) | 50S ribosomal protein | TSB |
| W8VJ01 | LsrB | 38.1 | 9.1 | 257 | 4 | 13 | P (9.84) | Autoinducer binding protein | TSB |
| W8V963 | TldD | 51.2 | 4.9 | 251 | 4 | 10 | C (9.26) | Microcin-processing peptidase | TSB |
| W8VCC3 | Lpp | 8.4 | 9.5 | 248 | 3 | 49 | OM (9.93) | Lipoprotein, intestinal colonization | TSB |
| W8V8D3 | Wzi | 55.7 | 5.5 | 242 | 4 | 10 | OM (9.52) | Polysaccharide export | TSB |
| W8V6H8 | HslU | 49.7 | 5.2 | 240 | 4 | 10 | C (9.97) | ATP-dependent protease ATPase | TSB |
| W8VNX2 | Map | 29.2 | 5.5 | 240 | 3 | 13 | C (9.97) | Cleaves methionine from peptides | TSB |
| W8V0X0 | YcfD | 42.6 | 4.6 | 238 | 3 | 13 | C (9.97) | Putative translation factor | TSB |
| W8V4F7 | RplB | 29.8 | 11.4 | 227 | 4 | 19 | C (9.97) | 50S ribosomal protein | TSB |
| W8VKB3 | SlyB | 15.2 | 9.4 | 225 | 3 | 32 | OM (9.92) | Membrane integrity, siderophore assembly | M63 |
| W8V5J5 | HflK | 45.5 | 5.9 | 224 | 3 | 13 | C (5.48) | NADP-specific glutamate dehydrogenase | TSB |
| W8VL55 | GdhA | 46.3 | 5.6 | 223 | 3 | 10 | C (9.97) | NADPH formation from glutamate | TSB |
| W8USH1 | RpsH | 14.1 | 9.8 | 221 | 3 | 26 | C (9.26) | 30S ribosomal protein | M63, TSB |
| W8VJG6 | Hypothetical | 36.3 | 5.3 | 220 | 3 | 11 | C (8.96) | Capsid protein | TSB |
| W8VKH7 | AdhP | 35.3 | 5.8 | 218 | 3 | 12 | C (9.97) | Oxioreductase | TSB |
| W8VFR2 | Frr | 20.6 | 6.9 | 216 | 4 | 29 | C (9.97) | Releases ribosomes from messenger RNA | M63, TSB |
| W8VGS8 | DsbA | 22.9 | 5.3 | 215 | 4 | 31 | P (10.0) | Disulfide bond formation | TSB |
| W8V696 | GmhA | 20.8 | 5.8 | 210 | 3 | 20 | C (9.97) | LPS biogenesis | TSB |
| W8VM98 | PotF | 41.6 | 8.5 | 210 | 3 | 11 | P (10.0) | Putrescine and polyamine transport | M63 |
| W8V8Z4 | FkpA | 29.4 | 9.2 | 207 | 3 | 12 | P (10.0) | Peptidyl-proline modification | TSB |
| W8VAM7 | LdhA | 36.3 | 5.5 | 206 | 3 | 12 | C (9.97) | Lactose fermentation | TSB |
| W8UY05 | AfuA | 39.5 | 5.5 | 204 | 3 | 12 | NA | Iron transporter | TSB |
| W8VC32 | GlnS | 63.7 | 5.4 | 203 | 4 | 10 | C (9.97) | Glutamine-tRNA ligase | TSB |
| W8VC05 | Pal | 19.7 | 7.4 | 203 | 3 | 20 | OM (10) | Serum-resistance, promotes inflammation | M63 |
| W8VLX5 | FabG | 25.5 | 6.2 | 196 | 3 | 18 | C (9.26) | Fatty acid elongation | TSB |
| W8VJ03 | DhaK | 38.3 | 4.7 | 195 | 4 | 18 | C (8.96) | Glycerone kinase | TSB |
| W8US00 | YhjJ | 57.1 | 5.6 | 195 | 3 | 10 | NA | Putative membrane metalloprotease | TSB |
| W8VP03 | CueO | 58.1 | 6.2 | 192 | 4 | 10 | P (10.0) | Cupredoxin | TSB |
| W8V703 | Hypothetical | 39.0 | 4.7 | 191 | 3 | 13 | C (8.96) | Major capsid protein | TSB |
| W8VCD2 | SorD | 34.1 | 5.7 | 191 | 3 | 14 | C (9.26) | Sorbose dehydrogenase | TSB |
| W8V972 | RpsI | 14.8 | 11.4 | 190 | 3 | 23 | C (9.97) | 30S ribosomal protein S9 | TSB |
| W8VIV6 | RplX | 11.3 | 10.7 | 186 | 3 | 20 | C (9.26) | 50S ribosomal protein | M63 |
| W8UZH6 | ManX | 34.8 | 5.9 | 182 | 3 | 12 | C (9.97) | Mannose transport | TSB |
| W8V864 | PurD | 46.4 | 4.6 | 182 | 3 | 11 | C (9.97) | Purine biosynthesis | TSB |
| W8VQG5 | MetB | 41.5 | 5.5 | 178 | 3 | 10 | C (9.97) | Cystathionine synthase | M63 |
| W8VIV9 | RplQ | 14.4 | 11.5 | 175 | 3 | 26 | C (9.26) | 50S ribosomal protein | TSB |
| W8VKE8 | YncE | 38.3 | 9.3 | 175 | 3 | 14 | NA | Unknown with periplasmic signal peptide | M63 |
| W8V917 | RpsM | 13.2 | 11.2 | 165 | 2 | 22 | C (9.97) | 30S ribosomal protein | M63 |
| W8UVI6 | BamC | 37.2 | 7.3 | 155 | 3 | 15 | OM (9.92) | Outer membrane protein assembly | M63 |
| W8VIQ1 | HldD | 34.9 | 4.7 | 149 | 3 | 11 | C (9.97) | LPS biogenesis | TSB |
| W8VLN3 | OsmE | 12.3 | 8.5 | 146 | 3 | 57 | NA | Osmotically-inducible protein | M63 |
| W8VD59 | DapD | 29.8 | 5.3 | 144 | 2 | 10 | C (9.97) | Lysine biosynthesis | TSB |
| W8V9H6 | DhaT | 41.4 | 5.9 | 143 | 3 | 11 | C (9.97) | 1,3-propanediol dehydrogenase | M63 |
| W8V3G8 | WrbA | 21.3 | 5.6 | 134 | 2 | 15 | NA | Hydroquinone reduction | TSB |
| W8VMN6 | GpmA | 28.3 | 5.6 | 127 | 2 | 13 | C (8.96) | Carbohydrate degradation, glycolysis | TSB |
| W8VJE0 | RplS | 13.1 | 11.1 | 123 | 2 | 23 | C (9.97) | 50S ribosomal protein | M63 |
| W8VD17 | YqjM | 39.5 | 5.9 | 107 | 2 | 12 | C (9.26) | flavin oxioreductase | TSB |
| W8UUQ2 | RplV | 12.2 | 10.7 | 106 | 2 | 19 | C (9.97) | 50S ribosomal protein | M63 |
| W8VIY1 | RplU | 11.5 | 10.3 | 104 | 2 | 21 | C (9.97) | 50S ribosomal protein | M63 |
| W8V4H4 | RpsK | 13.8 | 11.8 | 92 | 2 | 10 | C (9.26) | 30S ribosomal protein S11 | M63 |

Proteins from spent culture media were identified by liquid chromatography combined with tandem mass spectrometry (LC-MS/MS). The number of peptide hits was reduced to fit the stringent Paris Guidelines, which ensure that each peptide hit represents a unique peptide sequence, not just individual peptide modifications. The putative function for each protein was taken from annotations in the Uniprot database or when referenced from individual studies. The cellular localization of each protein was predicted using the software Psortb 3.0, which assigns a confidence estimate between 0 and 10 for each cellular location. Abbreviations: OM, outer membrane; CM, cytoplasmic membrane; C, cytoplasm; EX, extracellular; P, periplasmic; NA, unknown; LE, LB-exponential phase of growth; LS, LB-stationary phase of growth; RE, RPMI-exponential phase of growth; RS, RPMI-stationary phase of growth; ATP, adenosine triphosphate; MW, molecular weight; SC, sequence coverage.
